# Ecological-Health Risk Assessments of Heavy Metals (Cu, Pb, and Zn) in Aquatic Sediments from the ASEAN-5 Emerging Developing Countries: A Review and Synthesis

**DOI:** 10.3390/biology11010007

**Published:** 2021-12-21

**Authors:** Chee Kong Yap, Khalid Awadh Al-Mutairi

**Affiliations:** 1 Department of Biology, Faculty of Science, Universiti Putra Malaysia (UPM), Serdang 43400, Selangor, Malaysia; 2 Department of Biology, Faculty of Science, University of Tabuk, P.O. Box 741, Tabuk 71491, Saudi Arabia; kmutairi@ut.edu.sa

**Keywords:** ASEAN, heavy metals, sediments, ecological risks, human health risks

## Abstract

**Simple Summary:**

This study aimed to review and compile the concentrations of Cu, Pb, and Zn in the aquatic sediments of the ASEAN-5 countries published in the literature from 1981 to February 2021. The mean values of Cu, Pb, and Zn in aquatic sediments were found to be elevated and localized in high human activity sites in comparison to the earth’s upper continental crust and reference values. All reports (100%) showed the Zn ecological risk (ER) values were categorized as being between ‘low potential ER’ and ‘considerable potential ER’. Almost all Cu ER values (97.7%) showed similar ranges of the above two risk categories except for a few reports. Almost all reports (96%) showed Pb ER values categorized as between ‘low potential ER’ and ‘moderate potential ER’ except for a few reports. For the ingestion and dermal contact pathways for sediments, all non-carcinogenic risk (NCR) values for Cu, Pb, and Zn reflected no NCR. This provided evidence for the ASEAN-5 group of countries to be considered as being a new socio-economic corridor. Lastly, we claim that this study is currently the most up-to-date review on this topic in the literature.

**Abstract:**

The ASEAN-5 countries (Malaysia, Indonesia, Thailand, Philippines, and Vietnam) of the Association of Southeast Asian Nations as a group is an ever-increasing major economy developmental hub in Asia besides having wealthy natural resources. However, heavy metal (HM) pollution in the region is of increasing environmental and public concern. This study aimed to review and compile the concentrations of Cu, Pb, and Zn in the aquatic sediments of the ASEAN-5 countries published in the literature from 1981 to February 2021. The mean values of Cu, Pb, and Zn in aquatic sediments were elevated and localized in high human activity sites and compared to the earth’s upper continental crust and reference values. Based on 176 reports from 113 publications, the ranges of concentrations (mg/kg dry weight) were 0.09–3080 for Cu, 0.37–4950 for Zn, and 0.07–2666 for Pb. The ecological risk (ER) values ranged from 0.02–1077 for Cu, 0.01–95.2 for Zn, and 0.02–784 for Pb. All reports (100%) showed the Zn ER values were categorized as being between ‘low potential ecological risk’ and ‘considerable potential ecological risk’. Almost all Cu ER values (97.7%) also showed similar ranges of the above two risk categories except for a few reports. The highest Cu level (3080 mg/kg dry weight) was reported from a mine-tailing spill in Marinduque Island of the Philippines with ‘very high ecological risk’. In addition, drainage sediments in the western part of Peninsular Malaysia were categorized as Cu ’high potential ecological risk’. Almost all reports (96%) showed Pb ER values categorized as between ‘low potential ecological risk’ and ‘moderate potential ecological risk’ except for a few reports. Six reports showed Pb ER values of ‘considerable potential ecological risk’, while one report from Semarang (Indonesia) showed Pb ER of ‘very high ecological risk’ (Pb level of 2666 mg/kg dry weight). For the ingestion and dermal contact pathways for sediments from the ASEAN-5 countries, all non-carcinogenic risk (NCR) values (HI values 1.0) for Cu, Pb, and Zn reflected no NCR. The ER and human health risk assessment of Cu, Pb, and Zn were compared in an integrative and accurate manner after we reassessed the HM data mentioned in the literature. The synthesis carried out in this review provided the basis for us to consider Cu, Pb, and Zn as being of localized elevated levels. This provided evidence for the ASEAN-5 group of countries to be considered as being a new socio-economic corridor. Beyond any reasonable doubt, an ever-increasing anthropogenic input of HMs is to be expected to a certain degree. We believe that this paper provides the most fundamental useful baseline data for the future management and sustainable development of the aquatic ecosystems in the region. Lastly, we claim that this review is currently the most up-to-date review on this topic in the literature.

## 1. Introduction

According to the World Economic Outlook Database [1], the Association of Southeast Asian Nations ASEAN-5 countries are categorized as ‘Emerging and developing Asia’. The ASEAN-5 countries include Indonesia, Malaysia, Thailand, the Philippines, and Vietnam. According to Worldometer [2], as of 2020, populations of these countries are Indonesia (273.5 million), the Philippines (109.6 million), Vietnam (97.3 million), Thailand (69.8 million), and Malaysia (32.4 million).

The gross domestic product (GDP) in the ASEAN-5 countries in 2020 is presented in Table 1. With a 582.6 million population in total, the ASEAN-5 countries have the third largest population in the world (after China; 1442.7 million and India; 1388.3 million) and a GDP of USD 2.85 trillion (Table 1).

Many studies on sediment heavy metals (HMs) have been widely reported from the ASEAN-5 countries, including Malaysia [6,7,8,9,10], Thailand [11,12,13], Philippines [14], Vietnam [15,16], and Indonesia [17,18,19]. All the studies partly assessed the ecological risk (ER) of metals but not at all for the human health risk assessment (HHRA) of HMs.

People living in coastal regions and coastal reclamation areas should be aware of the HM exposure danger in estuarine wetlands. This is especially true for fishermen and consumers of local fish. The ecological and human health concerns of sediment-associated metals in large-scale estuary areas were investigated in this study since the ecological-human risks of HMs in the sediments had only recently been reported in the literature [20,21].

HM pollution in sediment is of severe concern, especially in developing countries, and it necessitates intensive monitoring to understand the current condition and to suggest potential remedial strategies [22]. During the years 1999 to 2009, Fang and Yang [23] examined data on Cu, Pb, and Zn in river sediments in Taiwan, China, Japan, India, and Vietnam. They found that anthropogenic activities such as home and industrial wastewater, as well as e-waste recycling, chemical, electric plating, and refining industries, were the main contributors of HM contamination. Haque et al. [24] examined several regulating parameters after reviewing more than 250 papers on metal–soil–plant interactions; Cu, Pb, and Zn were the metals studied. They summarized the HM pollution’s impact on the soil environment, plant physiology, and factors influencing individual metal uptakes.

### 1.1. Why Focus on Sediments?

Firstly, there are a number of publications in the literature on this topic. Based on papers published in academic journals from 1964 until February 2021, listed in the Scopus database, there were 8196 papers with paper titles that included ‘metal’ and ‘sediments’. In 2020 alone, there was a total of 493 papers. We expect that the number would be higher if the titles of the papers included ‘element’ and ‘pollution’. The number is also expected to be significantly higher if non-Scopus indexed journals were to be taken into account. These numbers indicated the importance of monitoring HMs in aquatic sediments. It is reasonable to believe that such monitoring studies would be increasing in the future.

Secondly, HMs in sediments can constitute secondary sources of contamination in aquatic ecosystems due to social-economic activities such as smelting, electroplating, leather making, and electronics, agriculture (fertilizers and pesticides), and aquaculture industries. All the above activities intensify the stresses on the water and the environment by producing large quantities of municipal [25,26], and industrial wastewater [8,27,28,29] that contain potentially toxic chemicals including HMs.

Thirdly, the sediment is the sink of potentially toxic HMs [30]. HMs are mostly delivered into the marine environment via rivers and estuaries, where they are frequently bound to particulate matters, which settle and are absorbed into the sediment. As a result, in aquatic ecosystems, surface sediments are the primary storage and sink for metals and contaminants [31,32].

### 1.2. Why Use the Ecological Risk Index?

First, based on Scopus data searched on 9 January 2021, the use of the potential ecological risk index (PERI), which was first proposed by Hakanson [33], received a total of 3682 hits. Hakanson [33] and Zhuang et al. [34] were the first and last citations, respectively. For Cu, Pb, and Zn, all these citations employed the same toxic response factor. This could be due to the fact that there is no more recent research in the literature. The background levels used in the quoted data from this paper, on the other hand, are different. The pre-industrial reference levels proposed by Hakanson [33], the upper continental crust (UCC) levels of the HMs proposed by Wedepohl [35], Turekian and Wedepohl [36], Taylor and McLennan [37], and Rudnick and Gao [38] were among the background levels used in the literature. The background values of Wedepohl [35] were used in this work for PERI determinations because they have garnered 3508 citations in Scopus so far (until 10 January 2021). Secondly, because of the variability and lack of comprehensiveness of HM risk estimates in surface sediments in the literature [39], in the present study we used the reported data of Cu, Pb, and Zn and re-analyzed them for ER values, which were then used to make accurate comparative interpretations. We also used the similar levels of background upper continental earth crust (UCC) by Wedepohl [35] of the three HMs besides the toxic response levels by [33]. Furthermore, the PERI was also based on Cu, Pb, and Zn.

### 1.3. Objectives of the Study

This paper compiled the data of Cu, Pb, and Zn in the aquatic sediments from SEA countries available in the literature from 1980 to January 2021. In this paper, the literature was reviewed and the data published by various authors were analyzed to assess the HM status of aquatic sediments of the ASEAN-5 countries, namely Malaysia, Indonesia, Thailand, Philippines, and Vietnam. Therefore, the objectives of this paper were (1) to review the three HM concentrations in the aquatic sediments from the ASEAN-5 countries, and (2) to determine the ER and human health risk assessment (HHRA) based on the reviewed data for effective comparative study and interpretation.

## 2. Methodology

### 2.1. Data Collection

**Figure 1:**
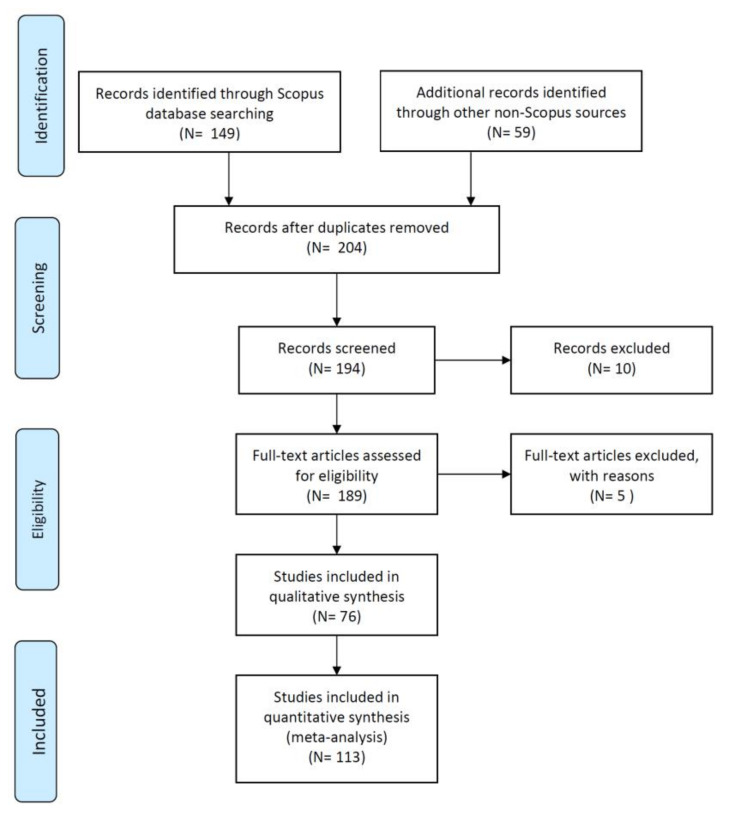
Flowchart of the Preferred Reporting Items for Systematic Reviews and Meta-Analyses (PRISMA) (adapted from Moher et al. [40]), used in the present study.

The current review research followed the systematic literature review (SLR) approach of Moher et al. [40] from the Preferred Reporting Items for Systematic Reviews and Meta-Analyses (PRISMA) to add to the existing body of knowledge on HMs in ASEAN-5 aquatic sediments. PRISMA is a reporting standard that is based on scientific evidence and can be used for critical evaluation. Figure 1 depicts the formal approach’s measures, which have been modified for this review work.

Due to Scopus being the most widely utilized database for conducting literature searches, it was employed for the analysis. Scopus is the largest dynamic reference information database that investigated writings that included logical diaries, novels, and gathering procedures [41] as of 29 April 2020. The evaluated papers comprised of the scholastic distributions on the topics of ‘Metals’, ‘Sediments’ and ‘Malaysia or Indonesia or Thailand or Philippines or Vietnam’ that were accessible on the Scopus bibliographic information base, chosen for its size and assortment of distributions.

On 1 February 2021, a survey of the literature based on the databases of Scopus and non-Scopus was conducted to arrive at a total of 208 published papers (149 Scopus indexed and 59 non-Scopus indexed papers) (Figure 1) where the keywords ‘Metals’, ‘Sediments’ and ‘Malaysia or Indonesia or Thailand or Philippines or Vietnam’ must be found in the title of the papers under the Scopus database. Due to duplicate publications, four papers were removed, with 204 papers remaining.

However, out of the 204 papers (Figure 1), a total of 15 papers were irrelevant and they were discarded from this review paper since the present paper focused on aquatic sediments including lakes, rivers, and coastal environment of the ASEAN-5 countries. Therefore, only 189 papers remained for evaluation in this review paper where 76 papers were used for the qualitative synthesis and 113 papers were included for the quantitative synthesis. The 76 papers used for the qualitative synthesis were Indonesia (26 papers), Thailand (13 papers), Vietnam (14 papers), Malaysia (17 papers), and the Philippines (6 papers).

The 113 papers included for the quantitative synthesis were relevant and representative of the study area in the ASEAN-5 countries. Most importantly, they were selected based on their presentations of data on Cu, Pb, and Zn in them screened and their contents were scrutinized. The quantitative synthesis was carried out by the calculations of the ER and the HHRA of Cu, Pb, and Zn based on the selected 113 papers, which consisted of 84 Scopus-indexed papers (74%), and 29 non-Scopus papers (26%). Overall, there were 15 papers from Indonesia (9 Scopus and 6 non-Scopus papers; 24 reports), 11 papers from Thailand (9 Scopus and 2 non-Scopus papers; 18 reports), 15 papers from Vietnam (13 Scopus and 2 non-Scopus papers; 24 reports), 65 papers from Malaysia (46 Scopus and 19 non-Scopus papers; 99 reports), and 7 papers from Philippines (7 Scopus paper; 11 reports) (Table 2).

### 2.2. Data Interpretation

#### 2.2.1. Ecological Risk Index (ERI)

Potential ecological risk index (PERI) was used to determine the potential risk of the HMs in the topsoil to the ecology. This PERI was proposed by Hakanson [33]. The tabulations of PERI were done in a series of formulas.

Firstly, the calculation of contamination factor (Cf) was based on the pollution of a single metal factor in Equation (1).

(1) $$\mathrm{Cf}=\frac{C_{s}}{C_{B}}$$

where C_s_ is the concentration of the metal in the topsoil. C_B_ is the background value of each metal in the topsoil. The present study used the background concentration in the earth’s upper continental crust (UCC) which are Cu (14.3 mg/kg), Pb (17.0 mg/kg), and Zn (52.0 mg/kg) based on Wedepohl [35].

Secondly, the calculation of ER, which is the potential ER of a single element, was calculated based on Equation (2).

(2) $$\mathrm{ER}=T_{R}\times \mathrm{Cf}$$

where T_R_ is the toxic response factor of a single element. The T_R_ values used in the present study are Cu = 5.00, Pb = 5.00, and Zn = 1.00, according to Hakanson [33]. According to Hakanson [33], the ER is divided into five categories: ‘low potential ecological risk’ (Er < 40), ‘moderate potential ecological risk’ (40 ≤ ER < 80), ‘considerable potential ecological risk’ (80 ≤ ER < 160), ‘high potential ecological risk’ (160 ≤ ER < 320), and ‘very high ecological risk’ (ER ≥ 320).

Lastly, the summation of all the ER values for each metal would result in the PERI value, which was calculated based on Equation (3).

(3) PERI=∑ER

According to Hakanson [43], there are four different classifications for PERI values: ‘low ecological risk’ (PERI < 150), ‘moderate ecological risk’ (150 ≤ PERI < 300), ‘considerable ecological risk’ (300 ≤ PERI < 600), and ‘very high ecological risk’ (PERI ≥ 600).

#### 2.2.2. Human Health Risk Assessment

Ingestion, inhalation, and skin contact are the three main routes via which humans are exposed to sedimentary HMs [135]. Coastal sediments, on the other hand, are found at the land–sea interface and interchange with coastal waters. Hence, the dosage from sedimentary inhalation was not computed. As a result, in this study, we measured the indices linked to sediment based on two main paths to determine risk assessment: ingestion and dermal contact [20,136].

Human health risk assessment (HHRA) of topsoils is generally utilized to measure non-carcinogenic risk (NCR) to humans using three exposure pathways namely ingestion, inhalation, and dermal contact. The HHRA technique was based on the US Environmental Protection Agency’s recommendations and the Exposure Factors Handbook [135,137,138,139]. For both children and adults, average daily doses (ADDs) (mg/kg day) of HMs were determined through ingestion (ADD_ing_), and dermal contact (ADD_der_) by using Equations (4)–(5) as shown below:

(4) $${\mathrm{ADD}}_{\mathrm{ing}}=C_{\mathrm{sediment}}\left(\frac{\mathrm{IngR}\times \mathrm{EF}\times \mathrm{ED}}{\mathrm{BW}\times \mathrm{AT}}\right)\times 10^{-6}$$

(5) $${\mathrm{ADD}}_{\mathrm{der}}=C_{\mathrm{sediment}}\left(\frac{\mathrm{SA}\times \mathrm{AF}\times \mathrm{ABS}\times \mathrm{EF}\times \mathrm{ED}}{\mathrm{BW}\times \mathrm{AT}}\right)\times 10^{-6}$$

where ADD_ing_, and ADD_der_ is the daily amount of exposure to metals (mg/kg day) through ingestion, and dermal contact, respectively. In this study, the NCR of HMs was assessed by using the hazard quotient (HQ) and hazard index (HI) [140,141]. The definition, exposure factors, and reference values used to estimate the intake values and health risks of HMs in topsoils collected from Peninsular Malaysia are presented in Table 3.

The HQ is the ratio of a metal’s ADD to its reference dose (RfD) for exposure pathways that are identical [137]. The RfD (mg/kg day) is the maximum daily dosage of metal from a certain exposure pathway that is considered as not posing a significant risk of detrimental consequences to sensitive individuals throughout their lives, including both children and adults. For Cu, the RfD (mg/kg day) values used in the present study were 4.00 × 10^−2^, and 1.20 × 10^−2^ for ingestion, and dermal contact, respectively. For Pb, the RfD (mg/kg day) values used in the present study were 3.50 × 10^−3^, and 5.25 × 10^−4^ for ingestion, and dermal contact, respectively. For Zn, the RfD (mg/kg day) values used in the present study were 3.00× 10^−1^, and 6.00 × 10^−2^ for ingestion, and dermal contact, respectively [141]. If the ADD was less than the RfD value (HQ 1), no adverse health impacts were expected, but if the ADD was greater than the RfD value (HQ > 1), there were likely to be harmful health impacts [137,139].

Hazard index (HI), which is the sum of the HQs in the two exposure paths, determines the NCR. [144,145,146]. A HI of < 1.0 was expected to show that there was no significant risk of non-carcinogenic effects. A HI of > 1.0 was expected to show that there was a possible occurrence of non-carcinogenic effects. The probability of non-carcinogenic effects has a positive connection with the increment of HI value [147]. The HI is calculated following Equation (6).

(6) $$\mathrm{HI}=\sum {\mathrm{HQ}}_{i}=\sum \left(\frac{{\mathrm{ADD}}_{i}}{{\mathrm{RfD}}_{i}}\right)$$

## 3. Results and Discussion

### 3.1. Heavy Metals in Sediments

The concentrations of Cu, Pb, and Zn reported from the ASEAN-5 countries as obtained from the literature are presented in Table 2. Based on 176 reports from 113 publications, the ranges of concentrations (mg/kg dry weight) were 0.09–3080 for Cu, 0.37–4950 for Zn, and 0.07–2666 for Pb.

**Figure 2:**
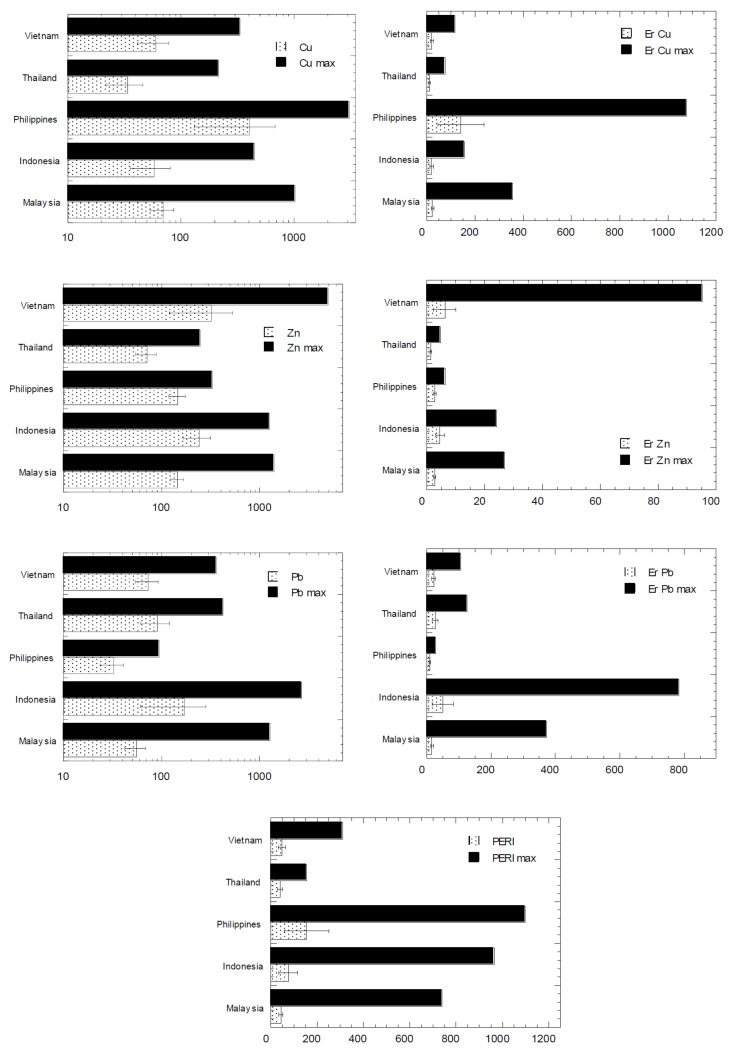
Overall statistics of values (mean ± SE, and maximum) of concentrations (mg/kg dry weight) of Cu, Zn, and Pb, and their ecological risks (Er) and potential ecological risk index from the ASEAN-5 countries in the present study.

**Figure 3:**
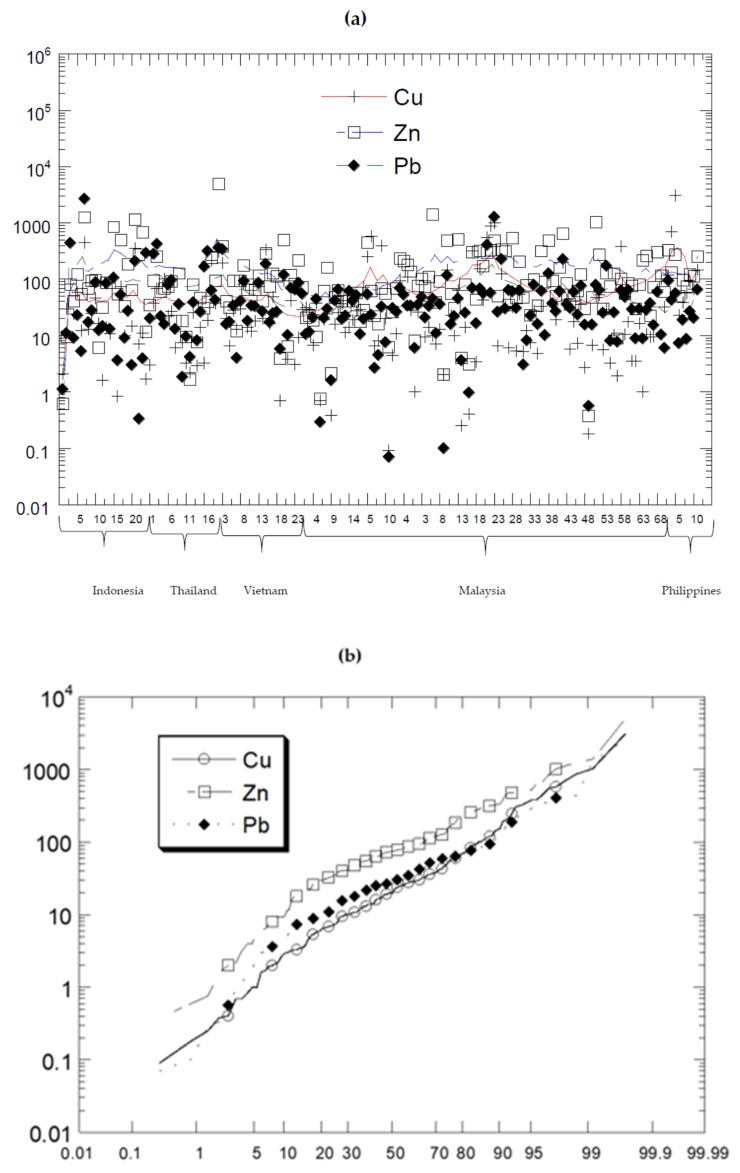
The distribution levels of Cu, Pb, and Zn were reported from (**a**) the ASEAN-5 countries, and (**b**) their probabilities of percentages for comparison. The log_10_ *Y*-axis represents the metal concentrations (mg/kg dry weight) for both (**a**) and (**b**).

The overall descriptive statistics of HM concentrations (mg/kg dry weight) in the aquatic sediments of the ASEAN-5 countries are presented in Table 4. For comparison, the distribution levels of Cu, Pb, and Zn reported from the ASEAN-5 countries are presented in Figure 2. The natural background levels of UCC by Wedepohl [35] for the metal distributions followed: Zn > Cu > Pb. From Table 4, the mean values of the three metals in Indonesia, Thailand, and Vietnam followed: Zn > Pb > Cu. However, Malaysia followed: Zn > Cu > Pb, while the Philippines followed: Cu > Zn > Pb. Overall, the probabilities of the percentages in Figure 3 indicated the overall metal distribution followed: Zn > Pb > Cu.

Therefore, the above metal distributions from Indonesia, Thailand, Vietnam, and the Philippines did not follow the UCC metal distribution by Wedepohl [35]. This indicated metal redistributions of the natural abundances of Cu, Pb, and Zn. This could be attributed to anthropogenic inputs of elevated Cu levels in the Philippines (mean: 57.8; 3.85 times higher than the Pb UCC level), and the elevated levels of Pb in Indonesia (Semarang, 2666; Jakarta Bay, 438), Thailand (Ao Ban Don and Pattani Bay; 424) and Vietnam (Rivers in Hanoi and Paddy field near Red River Delta, 361 and 340, respectively) (Table 2).

When compared to the concentrations of Cu, Pb, and Zn based on sediment quality guidelines and reference values (Table S1), the overall mean values of Cd, Pb, and Zn of the ASEAN-5 countries were all above those of the eight reference values.

### 3.2. Potential Ecological Risk Index

Based on 176 reports of the ASEAN-5 countries, the values of Cf, ER, and PERI calculated for the present study based on the cited concentrations of Cu, Pb, and Zn are presented in Table S2.

The overall ranges of concentrations, ER, and PERI of Cu, Pb, and Zn from the ASEAN-5 countries are presented in Figure 1. The ER values ranged from 0.02–1077 for Cu, 0.01–95.2 for Zn, and 0.02–784 for Pb (Table 1). The overall statistics of values (mean and maximum) of the concentrations of Cu, Zn, and Pb, and their ER and PERI from the present study are presented in Figure 2.

Almost all Cu ER values (97.7%) showed similar ranges of the above two categories except for a few reports. The highest Cu level (3080 mg/kg dry weight) was reported from a mine-tailing spill in Marinduque Island, Philippines, with ‘very high ecological risk’. In addition, drainage sediments in the western part of Peninsular Malaysia were categorized as Cu ‘high potential ecological risk’.

Almost all reports (96%) showed Pb ER were categorized as between ‘low potential ecological risk’ and ‘moderate potential ecological risk’ except for a few reports. Six reports showed Pb ER of ‘considerable potential ecological risk’, while one report from Semarang (Indonesia) showed Pb ER as ‘very high ecological risk’ (Pb level as 2666 mg/kg dry weight). All reports (100%) showed Zn ER were categorized as between ‘low potential ecological risk’ and ‘‘considerable potential ecological risk’.

Based on the country, the overall statistics of values of concentrations (mg/kg dry weight) of Cu, Zn, and Pb, and their Cf and ER from the present study are presented in Table 4.

### 3.3. Human Health Risk Assessment

The HHRA results due to Cu, Pb, and Zn exposures of sediments from the ASEAN-5 countries are presented in Tables S3–S5, respectively. Tables S3–S5 are based on 176 reports of the ASEAN-5 countries, showing the values of hazard quotient ingestion (HQ_ing_), hazard quotient dermal (HQ_dermal_), and HI of Cu, Pb, and Zn for children and adults from the present study. The overall statistics of values of HQ_ing_, HQ_dermal_, and HI of Cu, Pb, and Zn for children and adults from the present study are presented in Table 5.

It was found that all the NCR values showed HI values < 1.0 for Cu, Pb, and Zn. These values represented no NCR for the ingestion and dermal contact routes for sediments from the ASEAN-5 countries.

#### 3.3.1. Copper

For children Cu (S3), based on the mean values of the ASEAN-5 countries, the HQ_ing_ values ranged from 2.95E-05 to 1.01, and the HQ_dermal_ values ranged from 1.57 × 10^−7^ to 5.38 × 10^−3^. The HI values for children Cu ranged from 2.97 × 10^−5^ to 1.01. For adult Cu (S3), based on the mean values of the ASEAN-5 countries, the HQ_ing_ values ranged from 3.96 × 10^−6^ to 1.35 × 10^−1^, and the HQ_dermal_ values ranged from 4.02 × 10^−7^ to 1.37 × 10^−2^. The HI values for adult Cu ranged from 4.36 × 10^−6^ to 1.49 × 10^−1^. The Cu values of HQ_ing_ and HQ_der_ were higher in children than those in adults. Except for one report, the Cu HI values for both children and adults were lower than 1 in all the reports, indicating limited non-carcinogenic risk from Cu in the ASEAN-5 countries. The only Cu HQ_ing_ value > 1.0 was found in the Philippines (S5) at a mine-tailing spill on Marinduque Island (3080 mg/kg dry weight).

Cu has an average concentration of 50 parts per million in the earth’s crust. It is used in a variety of industrial and agricultural operations, and it can be discharged into the environment through a variety of sources, including mining, metal pipe, chemical manufacturing, and pesticide manufacturing [148,149]. Copper compounds are frequently employed in industrial and agricultural activities. As a result, increased Cu concentrations have been detected in some biosphere zones [150].

Though some HMs, such as Cu and Zn, are required by our bodies, large concentrations of these metals can be harmful [148,151]. Copper is also required for the formation of hemoglobin and plays an important role in enzymatic reactions. Copper plays a role in animal metabolism as well. However, excessive intake might be harmful to one’s health. Excessive Cu consumption poses major risks, including elevated blood pressure and respiration rates, kidney and liver damage, convulsions, cramps, vomiting, and even death [148].

#### 3.3.2. Lead (Pb)

For children, Pb (S4), based on the mean values of the ASEAN-5 countries, the HQ_ing_ values ranged from 2.59 × 10^−4^ to 9.87, and the HQ_dermal_ values ranged from 2.80 × 10^−6^ to 1.07 × 10^−1^. The HI values for children Pb ranged from 2.62 × 10^−4^ to 9.98. For adult Pb (S4), based on the mean values of the ASEAN-5 countries, the HQ_ing_ values ranged from 3.48 × 10^−5^ to 1.32, and the HQ_dermal_ values ranged from 7.14 × 10^−6^ to 2.72 × 10^−1^. The HI values for adult Pb ranged from 4.19 × 10^−5^ to 1.60. The Pb values of HQ_ing_ and HQ_der_ were higher in children than those in adults. Except for 10 reports from four countries, the Pb HI values for both children and adults were lower than 1 in all the other reports, indicating limited non-carcinogenic risk from Pb in the ASEAN-5 countries. The Pb HQ_ing_ values > 1.0 were from three reports (S3, S7, and S24) from Indonesia, three reports (S2, S3, and S17) from Thailand, two reports (S2 and S3) from Vietnam, and two reports (S21 and S23) from Malaysia.

Han et al. [152] conducted a bibliometric analysis of HM health risks and impacts to obtain an overview of HM health risk levels, sources, and control strategies in diverse regions throughout the world from 1989 to 2018. Over the last 10 years, there has been a considerable increase in concern about HM dangers and repercussions, particularly in China and other developing countries, according to the findings.

Pb is a naturally occurring metal with a concentration of 15–20 mg/kg in the earth’s crust [153]. Lead (Pb) is a very toxic HM that is non-biodegradable and rapidly accumulates in the human body [154]. It harms blood circulation, the central nervous system, the liver, and the kidneys in humans [154]. Paint, pesticides, smoking car emissions, coal burning, and mining are all sources of Pb contamination in the ecosystem. Lead is utilized in a variety of products, including batteries, ammunition, metal items like solder and pipes, and X-ray shielding devices, all of which cause health concerns [154]. Pb exposure has a major public health impact in developing countries, but only a few of these nations have implemented laws and legislation to effectively fight the problem [155].

Pb-related intellectual deficiencies have been documented at blood lead levels considerably below 10 g/dL in an international pooled investigation, which is likely to be of greatest concern to children under the age of 10. In the case of adults, several credible population studies have found a link between blood Pb and death risk [156].

#### 3.3.3. Zinc

For children, Zn (S5) based on the mean values of the ASEAN-5 countries, the HQ_ing_ values ranged from 1.62 × 10^−5^ to 2.16 × 10^−1^, and the HQ_dermal_ values ranged from 1.29 × 10^−7^ to 1.73 × 10^−3^. The HI values for children Zn ranged from 1.63 × 10^−5^ to 2.18 × 10^−1^. For adult Zn (S5), based on the mean values of the ASEAN-5 countries, the HQ_ing_ values ranged from 2.17 × 10^−6^ to 2.90 × 10^−2^, and the HQ_dermal_ values ranged from 3.30 × 10^−7^ to 4.42 × 10^−3^. The HI values for adult Zn ranged from 2.50 × 10^−6^ to 3.34 × 10^−2^. The Zn values of HQ_ing_ and HQ_der_ were higher in children than those in adults. All the Zn Pb HI values for both children and adults were lower than 1 in all the reports, indicating limited non-carcinogenic risk from Zn in the ASEAN-5 countries.

Many biochemical processes and physiological activities of living tissues are regulated by zinc. Excess Zn causes a variety of health issues, including stomach sickness, skin irritations, cramping, vomiting, and anaemia [151]. Brass plating, wood pulp manufacturing, ground and newsprint paper manufacture, steelworks with galvanizing lines, and Zn and brass metal works are examples of human activities and industrial sources of Zn. In various waste streams mentioned in the literature, Zn contents ranged from less than 1 to more than 48,000 mg/L. Sediment entrainment, agricultural operations, groundwater intrusion, or a combination of these sources release Zn into the environment [151].

### 3.4. Exposure Behaviors of Heavy Metals

For Cu (Table S3), in children, the percentages of HQ_ing_ to HI were 99.47% while HQ_dermal_ to HI was 0.53%. In adults, the percentages of HQ_ing_ to HI were 90.8% while HQ_dermal_ to HI was 9.21%. For Pb (Table S4), in children, the percentages of HQ_ing_ to HI were 99.9% while HQ_dermal_ to HI was 0.10%. In adults, the percentages of HQ_ing_ to HI were 83.0% while HQ_dermal_ to HI was 17.0%. For Zn (Table S5), in children, the percentages of HQ_ing_ to HI were 99.2%, while HQ_dermal_ to HI was 0.79%. In adults, the percentages of HQ_ing_ to HI were 86.8% while HQ_dermal_ to HI was 13.2%.

It was shown that the two different exposure pathways of Cu, Pb, and Zn for children and adults diminished in the following order: ingestion > dermal contact. The contributions of HQ*_ing_* to HI (total risk of non-carcinogenic) were the highest for Pb (99.9%) for children, while the lowest was found in the Pb (83.0%) for adults. The contributions of HQ*_dermal_* to HI (total risk of non-carcinogenic) were the highest for Pb (17.0%) for adults, while the lowest was found in the Pb (0.10%) for children. This emphatically showed that ingestion was the fundamental exposure pathway to undermine human health. Based on a report by Qing et al. [141] in Anshan, China. the highest Cd contribution (75.2%) to HI for adults was found in HQ_dermal_.

Comparison of the HI values for children and adults, found that children had a higher risk of NCR from HMs in the ASEAN-5 countries’ sediments, which is consistent with what has been observed for the Red Sea countries [148]. If HI 1, the exposed population would not display obvious detrimental health impacts, according to the USEPA [136]. The child group was exposed to a higher risk of negative health outcomes due to the pollutants’ influence. According to the findings, cutaneous absorption of pollutants was the primary reason of the child group’s risk. The increase in non-carcinogenic health risk was closely proportional to the human body’s exposed skin areas. Children are more susceptible to environmental contaminants because of their behavior and physiology. Pica behavior and hand or finger sucking are common in children, resulting in a greater NCR than in adults. They exhibit increased hand-to-mouth activity, respiration rates, and gastrointestinal absorption of some drugs [157]. The literature [156,157] has shown that HMs lead to greater health concerns to children than to adults.

### 3.5. Thailand

Thirteen qualitative papers from Thailand were not included in the calculations of ERI and HHRA [11,157,158,159,160,161,162,163,164,165,166,167]. The exclusions were mainly due to not all the three metals (Cu, Pb, and Zn) being analyzed and reported in these papers.

#### 3.5.1. The Gulf of Thailand

Worakhunpiset [168] compiled the findings of investigations on trace element levels in marine sediments from Thailand’s Gulf. Unsustainable land-based pollution produced by population increase, urbanization, and industrialization along the coastline is the primary source of marine environmental deterioration in the Gulf of Thailand [168]. The maximum values of Pb and Zn in maritime sand were 79.8 mg/kg and 800 mg/kg, respectively, among all the tested values [169]. Geographical spatial and temporal variations in HM levels had been reported to be related to anthropogenic activities such as tourism, agriculture, urbanization, aquaculture, industry, and port operations [168].

Previously, Polprasert [164] reported that HM contamination in the Chao Phraya River estuary revealed substantial accumulations of Cd, Cu, Cr, and Pb in the water near the river mouth, which could have long-term effects on the aquatic environment through HM precipitation to the bottom sediments and HM bio-accumulation and bio-magnification in various food chains. Even though the Thai government has implemented laws and regulations aimed at pollution control, the Upper Gulf of Thailand’s environmental quality remains a source of contention, necessitating scientific investigations [169]. The Chao Phraya River carries significant volumes of domestic (from Bangkok’s 10 million residents), aquaculture (with total aquaculture output of 7105 t annually (DoF, 2017)), agricultural, and industrial wastes into the Gulf of Thailand, either partially treated or untreated (PCD, 2019).

Hungspreugs and Yuangthong [160] reported that sediment cores from the Chao Phraya River mouth region were enriched with Cd and Pb at the top end of the cores, with these layers corresponding to the dated sediment cores [170]. For Cr, Cu, and Zn, no enrichment was discovered. The light pollution stage near the upper Gulf of Thailand and the coastal region close to Songkhla Province had been reached due to anthropogenic activities, according to Liu et al. [60].

According to Wijaya et al. [62], the total levels of Pb (62.6 ppm), Zn (240 ppm), As (27.2 ppm), Fe (16,636 ppm), and Mn (419 ppm) in the Chao Phraya River sediments were higher than in the Sumida River, suggesting a strong anthropogenic impact in Bangkok. According to Windom et al. [166], sediment budgets suggested that only a small portion of the sediment supplied to the Upper Gulf by the major rivers was subsequently transferred to the Lower Gulf.

Mingkhwan and Worakhunpiset [55] measured the amounts of Cd, Cr, Cu, Ni, Mn, Pb, and Zn in river and stream sediment collected in industrial estates in the Uthai and Bangpa-in Districts of Phra Nakhon Si Ayutthaya Province following the 2011 floods. They found that metal levels in sediment and fish samples were within the published guidelines. Strong metal-enriched concentrations (Hg, Cd, Zn, Ni, and Pb) were found in sediments of the Chao Phraya River Mouth between 2017 and 2019, according to Asokbunyarat and Sirivithayapakorn [157]. The elevated levels of HMs in sediments of the Chao Phraya River Mouth might be caused by weathering of rocks, sediment transport, and deposition within the Chao Phraya River Basin. This was due to the high freshwater inflow and discharge of low concentrations of HMs in processed industrial effluent to the Chao Phraya River.

#### 3.5.2. Other Rivers and Aquatic Ecosystems

HMs were discovered in the Mae Klong river’s coastal system and estuarine zone by Censi et al. [158]. They discovered a lithogenic component that influences the composition of coastal waters and suspended particles. Pengthamkeerati et al. investigated the status and seasonal change of HMs in surface sediment at Don Hoi Lot, a site in the Mae Klong estuary (2013). All the HMs detected in the sediments showed lower quantities than in the other surrounding estuaries, except for Zn.

Cheevaporn et al. [159] proposed that diagenetic remobilization and release of these metals from the sediments of the Bang Pakong River Estuary into the overlying water might lead to their subsequent redeposition into the sediments. The accumulation of HMs in the coastal sediments along with Chonburi to Pattaya, Chonburi Province, showed no major hazards, according to Khuntong et al. [161]. The levels of certain HMs in stream sediments from the middle part of the Songkhla Lake Basin were mainly controlled by anthropogenic activities, according to Ladachart et al. [162], and did not exceed the Dutch standard values.

Potipat et al. [58] revealed that the coastal area of Chanthaburi Province was unpolluted and not enriched by HMs (Pb, Cd, Ca, Fe, Cu, and Zn). Pb was found at high concentrations of 386 and 557 mg/kg in the dockyard and Pattani River-mouth sampling sites, respectively, according to Sowana et al. [165]. These findings revealed that using coastal lands without proper planning and management had negative impacts on the soil, especially in terms of sediment contamination.

### 3.6. Vietnam

Fourteen papers from Vietnam were not included in the calculations of ER and HHRA [23,25,27,68,171,172,173,174,175,176,177,178,179,180]. The exclusions were mainly due to not all the three metals (Cu, Pb, and Zn) being analyzed and reported in these papers.

#### 3.6.1. Northern Vietnam

In Hanoi, Vietnam, industrial and urban wastewater is dumped directly into rivers. Ho and Egashira [63] found that total HM concentrations ranged from background levels to over the maximum permissible levels to crop growth in sediments collected from different sites along three rivers in Hanoi City’s developed and densely populated area. They concluded that total HM concentrations were site-specific and appeared to be higher in areas with manufacturing companies.

The sediments of two rivers in Hanoi City (To Lich and Kim Nguu rivers) were extensively polluted with HMs, according to Huong et al. [27]. The sort of manufacturing plants placed along the rivers was directly tied to the high HM levels. As these two rivers are the primary supply of irrigation water for suburban agricultural land and feed water for fish farming ponds, this has sparked popular outrage. Ho et al. [181] investigated the speciation and mobility of As, Cu, Mn, Pb, and Zn in sediments at the Cam River mouth as a function of depth. They discovered that sediment-bound Pb and Mn dominated in the reducible and acid-soluble fractions, respectively, while Cu and Zn were fairly uniformly distributed across the four extracted fractions.

In the largest estuary ecosystem in northern Vietnam, Thinh et al. [179] found higher concentrations of As and HMs in river sediments and top-layers of mangrove forest soil cores in varied land uses. They speculated that increased harmful metal levels in the ecosystem could be the result of extensive human activities upstream of the Red River in recent decades.

Ho et al. [66] found that HM (containing Cu, Pb, and Zn) levels grew fast by two times or more in the Cam River mouth (Haiphong Province) from 1954 to 1975, then remained essentially steady from 1975 to 2008. The contamination of the Cam River mouth by HMs and As was largely due to anthropogenic operations in Haiphong Harbor and industrial zone. Nguyen and Volkova [173] discovered that 1.76–16.3 percent of the flow of HMs accumulated throughout the year in the Red River Delta, with the highest accumulation rates for Pb, Fe, and Zn. Nguyen et al. [69] found that at the upstream of the Red River, the enrichment factors of Cu, Cd, Pb, Ni, and Zn in sediments were significantly higher. Cu and Pb were found to be among the most prevalent contaminants in the Red River, with quantities ranging from moderate to severe pollution levels.

In the Red River Delta, Phuong et al. [28] found Cu, Pb, and Zn levels in the surface layer of a paddy field near a Cu casting village’s wastewater channel exceeded the limits allowed for Vietnamese agricultural soils. Near a fertilizer facility and an industrial zone, they found high levels of Zn collected in the surface soil of rice fields.

Hoai et al. [70] discovered that the concentrations of HMs (Cu, Zn, Pb, As, and Cd) in the sediments of tidal flats in northern Vietnam (Quang Ninh to Ninh Binh) provinces increased over time. Cu, Pb, and As concentrations in sediments from the center to the south surpassed the ISQG standards. The impacts of land-ocean interaction mechanisms and human activities were responsible for these findings.

#### 3.6.2. Halong Bay

In Halong Bay, Dang Hoai et al. [71] reported that the concentrations of Cu, Pb, and As were higher than the levels in the Interim Sediment Quality Guide (ISQG) of the coastal site, but lower than the levels in the ISQG of the marine site. This was because the area around the bay attracted several economic activities. The development of coal mining, shipping, aquaculture, and tourism, has led the region’s economy to develop rapidly [71,182].

Only As was of concern among the HMs examined, according to Ho et al. [181], while Cd, Co, Cr, Cu, Mn, Ni, Pb, and Zn appeared to represent their background concentrations in Cua Ong Habor sediments (Ha Long Bay). This is because natural processes including weathering and bedrock erosion are the primary sources of HMs in the sediments near Cua Ong Harbor. Ho et al. [67] discovered that HMs were derived from natural bio/geochemical processes and had low potential mobilities in the Cua Luc Estuary and Ha Long Bay. Thanh-Nho et al. [177] proposed that most HMs in the Can Gio Mangrove were of natural origins, accumulated in the mangrove primarily as oxyhydroxides originating from upstream lateritic soils. This was supported by the high proportion of metals in the residual fraction of HMs (>50%).

#### 3.6.3. Central Vietnam

There was a scarcity of published data on total metal levels in coastal sediments from Central Vietnam, especially Khanh Hoa province. Romano et al. [15] found that the levels of Li, Mn, Ni, V, Pb, Cr, Zn As, Cd, U, and Hg in sediments from the coastal lagoons Thuy Trieu and Dam Nai were higher than the calculated metal levels. The HMs in water and sediment samples collected from the Cai River estuary and Nha Trang Bay in 2010 at eight locations along the salinity gradient (ranging from 0–36 ppt) were recorded by Koukina et al. [16]. However, there was no mention of HM contamination.

In 2008, sediment cores were taken from nine central Vietnamese coastal lagoons (Lang Co, Truong Giang, An Khe, Nuoc Man, Nuoc Ngot, Thi Nai, O Loan, Thuy Trieu, and Dam Nai). The metals (Al, Cd, Cr, Cu, Fe, Hg, Li, Mn, Ni, Pb, V, U, and Zn) were generally low, according to Romano et al. [15], except for As, which often exceeded ERL standards. They concluded that the character of rocks and soils in the watersheds influenced the metal concentrations in the lagoon sediments.

#### 3.6.4. Southern Vietnam

Costa-Böddeker et al. [25] found that total HM levels in the Thi Vai Estuary and the Can Gio Mangrove Forest were lower than expected for this heavily industrialized location. Costa-Böddeker et al. [183] discovered that upstream Ni levels were above the likely impact level (PEL), indicating a possibility of unfavorable biological impacts. However, when past land-use impacts were taken into account (such as untreated wastewater discharge, intensive agricultural activities, tanning operations, and so on), pollutant concentrations in the Thi Vai Estuary were lower than expected, owing to dilution by high sedimentation rates and tidal hydrodynamics [25,183].

Costa-Böddeker et al. [171] discovered that Cr and Cu were over the threshold effect levels (TEL), whereas Ni was above the probable effects level (PEL), indicating the possibility of detrimental biological consequences. Point sources (recent industrialization and land-use changes), air pollution, and erosion were identified as the key causes of the increased HM level. Dung et al. [26] previously found minimal Cr, Cu, and Pb contamination in the Can Gio district. Under acidification, the risk of Mn, Zn, and Ni was estimated to be medium to extremely high. Thanh-Nho et al. [177] found that trace metal concentrations in the dissolved and particulate phases at flood tides in the Can Gio mangrove were in the same range as those recorded in the Can Gio Estuary.

Although the metropolitan region was highlighted as the major contributor for metal(oid) emissions, Strady et al. [176] revealed that the levels of metal contamination along the Saigon River looked to be moderate according to Vietnamese and WHO guidelines. This was due to the effects of wastewater and industrial discharges besides the water geochemistry along the river continuum. Later, Nguyen et al. [175] stated that the concentrations of 11 metals and metalloids in the sediment of the Saigon River varied with season and space and that three primary pollution sources namely from the Saigon River basin, inside Ho Chi Minh City, and the lowland contributed to enriching the HMs in the River sediment.

Only one area with relatively significant Cu enrichment was found in the seagrass beds off the coast of Khanh Hoa, according to Nguyen et al. [174]. Cu readings at My Giang and Pb values at Thuy Trieu, in particular, were in the intermediate risk category. As a result, two of the eight areas could be exposed to elevated Cu and Pb levels. In the southern coast, Tu et al. [180] reported that HMs (Cd, Pb, Hg, and As) levels in all samples were lower than the Vietnamese regulation levels and the probable effect levels in the CCME guideline.

### 3.7. Indonesia

Twenty-six papers from Indonesia were not included in the calculations of ERI and HHRA [184,185,186,187,188,189,190,191,192,193,194,195,196,197,198,199,200,201,202,203,204,205,206,207,208]. The exclusions were mainly due to not all the three metals (Cu, Pb, and Zn) being analyzed and reported in these papers.

Based on the publications between 1986 and 2008, Arifin et al. [209] summarized that toxic HMs were one of the widespread contaminants in Indonesian coastal waters.

#### 3.7.1. Jakarta Bay

Hosono et al. [49] investigated the 210Pb geochronology, HM concentrations (Zn, Cu, and Pb), and stable Pb isotope ratios (206Pb/207Pb) of three sediment cores taken from Jakarta Bay from 1900 to 2006 to unravel the history of HM contamination. They discovered that anthropogenic metal deposition in the sediments began in the 1920s and increased dramatically from the 1970s until the end of the 1990s, based on chemical and isotopic investigations. Zn and Pb accumulation rates in the coastal industrialised region were steady or declined from the end of the 1990s to 2006, while HM pollution is still a major problem in terms of environmental preservation and protection in Jakarta Bay. Williams et al. [44] found increased levels of Zn, Pb, Cu, and Ni in the Jakarta City Harbor, particularly in the center of the bay and to the east and west of the harbor, near to the bay’s shoreline. The geographic patterns of HMs were consistent with a prior investigation that showed substantially greater Pb and Zn concentrations in the eastern section of Jakarta Bay [44].

Anthropogenic causes affected Zn, Ni, Pb, and partly Cu, according to Sindern et al. [50], primarily in central Jakarta City. The data showed a wide range of local emission sources, including metal industry, fertilizers, and untreated animal feces, and street dust. Riani et al. [199] aimed to examine the effect of HM pollution on green mussels cultured in Muara Kamal Waters, Jakarta Bay over seven months. Their results showed that the HM concentrations in the sediment exceeded the quality standard at Muara Kamal.

#### 3.7.2. Java

In the Garang (Central Java) watershed, Haeruddin et al. [195] assessed the HM concentrations in the sediments and traced the distribution. Pb was found to have the highest concentration, followed by Cr and Cu, although Cd and Zn were found to be below detection limits. The Kreo River has the highest Pb values and the Banjir Kanal Barat River had the lowest. The Garang River had the greatest Cu contents, whereas the Kreo River had the lowest. According to the sediment pollution index, the sediments of the Garang watershed were not contaminated by HMs.

Isworo and Oetari [51] reported that the HM concentrations in the sediment collected from marine waters of Tanjung Jati Jepara (Central Java province) had values that were below the standards. But this could allow the process of HM biomagnification dam to occur, which, if continued in the long run, might be hazardous to marine ecosystems and the health of nearby communities. Widianarko et al. [43] measured the concentrations of Cd, Pb, Cu, and Zn in Semarang’s coastal urban sediments (Central Java). From 101 sites evaluated in the larger Semarang area, 51 were unpolluted, 36 were mildly polluted, 9 were polluted, and 5 were extremely polluted, according to this classification. Cu, Zn, Pb, and Cd contents in coastal silt in Usman Janatin Street (Semarang) were determined by Almiqrh et al. [184].

The levels of eight HMs (Cr, Cu, Fe, Mn, Ni, Ti, V, and Zn) in surface sediments from Segara Anakan Nature Reserve (SANR), a mangrove-fringed lagoon in the Cilacap coastal area, were determined by Syakti et al. [202]. Cr, Ni, and Zn concentrations identified in SANR sediments might induce harmful effects to occur throughout a wider variety of species in refinery site stations, according to the evaluation.

Sudarningsih et al. [201] collected sediment samples from Citarum River (West Java Province). They assessed the usefulness of magnetic methods in detecting HM pollution in the sediment samples. According to geochemical tests, all water and sediment samples contained Fe, Cd, Co, Ni, Pb, Zn, As, Hg, and Mn. Although there was a negative link between HM concentration and magnetic parameters, the HM levels varied sporadically in sediment samples.

Lestari et al. [197] collected surface sediment samples from 27 sites in Cirebon coastal waters (West Java) in April 2017. They determined metal fractionation to evaluate bioavailability in sediments from Cirebon coastal waters. The results showed that the metals Cu, Ni, and Zn originated from anthropogenic sources.

Based on the sediments collected from Bomo coastal water (East Java) in July 2018, Dewi et al. [190] showed that there was no significant difference between Pb, Cd, and Hg concentrations at all sites (*p* > 0,05). Furthermore, in general, concentrations of all HM had exceeded the quality standards for marine biota.

During the spring and neap tidal periods in Popoh Bay, Yona et al. (2018a) tested varying amounts of Pb and Cd in the water and sediment (Tulungagung, southern part of Java Island). Due to the higher input of Pb from oil pollution by boats and fishing operations, the concentration of Pb was higher than the concentration of Cd in both spring and neap tides. Water movement during spring and neap tides had a substantial effect on the HM distribution, according to their research. Yona et al. [207] investigated the geographic distribution of HM in four coastal locations in southern Pacific Pacitan (South Java). Pb and Hg levels were found to be significantly higher in Pantai Soge, while Cd levels were considerably higher in Pantai Pancer. Overall, HM concentrations in the research areas were modest; nevertheless, significant activities in the coastal areas of southern Pacitan could contribute to HM pollution, necessitating attention.

Harmesa and Cordova [193] evaluated the state of the Dasun estuary (Central Java) based on the accumulation of Cr, Cd, and Pb in sediments. Even though the concentration of Cd was rather high and was thought to originate from batik industry waste, the Dasun estuary was confirmed to be safe for the living habitat. Lestari et al. [196] collected Cu, Ni, and Zn from 25 typical sites in Banten Bay (West Java) in April 2016. The percentages of Cu, Ni, and Zn were primarily deposited in the residual fraction of overall concentrations, according to the results.

#### 3.7.3. Sumatra

Irzon et al. [194] investigated the HM concentrations in six tin tailings and two soils from Singkep Island concerning environmental risk. They found higher environmental problems in the sediments collected from the primary wastes.

The contents of Zn and Pb in surface sediments collected from 23 monitoring locations in Dumai coastal waters were determined by Amin et al. [210]. The resistant fraction dominated metal concentrations at 87% and 74% for Zn and Pb, respectively. They found anthropogenic Pb imports occurred in more sampling sites. This could be due to a mixture of factors, such as a big population, untreated home, and industrial waste discharges, shipping activities, and city run-off. The amounts of Cd, Cu, Pb, Zn, Ni, and Fe in Dumai coastal waters were comparable to other locations of the world, according to Amin et al. [19].

Amin et al. [52] showed that the distribution of HM concentrations in seawater and sediment on Kundur Island’s northwest coast followed virtually identical trends, with higher concentrations recorded in stations adjacent to mining activities. The PERI result suggested that the tested area was still at low ecological risk due to nearby mining operations. Karina et al. [195] analyzed the Mn, Pb, Cu, and Cd levels in fluvial sediments affected by coal spill water in Lampuuk, Aceh Province, collected in December 2018. They found that Pb content in the sediment was still under the threshold.

#### 3.7.4. Sulawesi

During the wet and dry seasons, Najamuddin et al. [198] measured Pb and Zn concentrations in surface sediments at 17 stations in riverine, estuarine, and marine settings in the estuary of the Jeneberang River (South Sulawesi). They discovered that HM concentrations were affected by the season and that Pb and Zn came primarily from rivers and natural sources.

Samawi et al. [200] collected sediments from four estuarine waters from the west coast of South Sulawesi and analyzed them for Pb, Cd, and Cu. This study showed that the bioavailability of HMs Pb, Cd, and Cu in the estuary waters from the west coast of South Sulawesi was considered low. The bioavailability of HMs in sediments was as follows: Cd > Pb > Cu.

Tongggiroh et al. [205] investigated the origin, level, and distribution of HMs in coastal sediments of Parepare Bay (South Sulawesi), which is home to a harbor, tourism, and a landfill. They discovered that the concentration of various non-earth sources resulted in an enrichment factor (EF) of Pb > 1. Armid et al. [186] studied HM pollution in the surface sediments of Kendari Bay (Southeast Sulawesi), suggesting that the Kendari Bay surface sediment was regarded as an unpolluted area by HMs such as Cu and Zn). According to Asaf et al. [187], the metal concentration in the coastal sections of Sangihe Island (Talengan Bay, Manalu, and Dagho Bay; North Sulawesi) was below the background value. Cu, Pb, and Zn levels in Makassar’s coastal water showed that it was still unpolluted, according to Bohari and Palutturi [188].

#### 3.7.5. Other Coastal Waters

Tanjung et al. [204] collected sand samples from six locations along the coast of Mimika Regency (one of Papua Province’s regencies) and analyzed them for HMs. The coastal waters of Mimika Regency were classified as low pollution and of low potential ER. As a result, Pb, Cu, Cd, and Hg were not found in the coastal waters of the Mimika Regency.

### 3.8. Malaysia

Seventeen papers from Malaysia were not included in the calculations of ERI and HHRA [6,90,211,212,213,214,215,216,217,218,219,220,221,222,223,224,225]. The exclusions were mainly due to not all the three metals (Cu, Pb, and Zn) being analyzed and reported in these papers.

Shazili et al. [220] summarized the sources of HM pollution in Malaysia, stating that they came from manufacturing, agricultural, sewage, and motor vehicle emissions. Pb and Zn contaminations were found in the Juru and Langat river sediments. The concentrations of Zn and Pb in coastal sediments off Juru, Penang, and in the Johor Straits were two and three times greater than the global shale values, according to the researchers. The usage of leaded gasoline was assumed to be the source of Pb contamination. Metal concentrations in sediments from the Malacca Straits and the South China Sea were similar to those found in worldwide shale. Salam et al. [113] found that Pb, Zn, and Cu in the Perak River came primarily from natural sources, with minimal input from human activity.

A recent review by Yunus et al. [226] revealed the different concentrations of pollutants categorized as (1) low, (2) moderately, and (3) chronically contaminated areas from HMs. From publications between 2000 and 2020, the anthropogenic inputs of HMs into the coastal environment of Peninsular Malaysia have shown a continuously increasing trend and these pollution studies are increasing yearly. However, there are valid reasons to suspect increasing amounts of HMs in the coastal sediments based on earlier speculations by Ismail et al. [96]. Due to increasing and rapid industrialization and urbanization, negative impacts have been afflicted on the health of the aquatic system.

#### 3.8.1. West Coast of Peninsular Malaysia

The west coast of Peninsular Malaysia (PM) is more populous than the east coast of PM and East Malaysia. Khodami et al. (2017) reported the PERI of HMs indicated that the Bayan Lepas Free Industrial Zone of Penang was at low risk. Earlier, due to ocean disposal and land reclamation, Sivalingam (1984) reported the daily input of Cu, Pb, and Zn as 3.44 × 10^2^ (kg/day), 1.29 × 10^4^ kg/day, and 2.46 × 10^3^ kg/day, respectively, into the marine ecosystem of the Western Channel by the Penang sewage ocean outfall. It was evident that the input of all these metals was very high. As the Western, North, and South Channels are flushed only by diurnal currents then ‘these metals are presumably being deposited in the sediments and so potentially assimilated within the marine food web.

Pb concentrations in the coast of Tanjung Karang (Selangor) and off Juru (Penang), according to Wood et al. [115], suggested some enrichment above the natural world value (in shale). Idriss and Ahmad [223] studied Cu, Cd, and Pb from the Juru River, Penang, Malaysia. Yusoff et al. [128] studied the HM contents in copepods, waters, and sediments from the unpolluted Gula estuary, and the polluted Juru estuary, off an industrial area in Penang. Malaysia. They found that the copepods and sediments from the Juru estuary contained significantly higher (*p* < 0.05) concentrations of Zn, Cu, Pb, and Cd compared to those from the Gula estuary. Redzwan et al. [77] found that quantitative evaluation using the HM enrichment factor was within the degree of background concentration in mangrove areas in Negeri Sembilan, Melaka, and Pahang.

Abubakar et al. [114] reported there were elevated concentrations of Cd, Cu, Pb, Zn, and Ni in the sediments of the Sungai Puloh mangrove area. Pollution by HMs in Sungai Puloh was caused by both anthropogenic and natural activities. The main sources of HM contamination in Port Klang, according to Sany et al. [8,29], were industrial effluent and port activities. According to Shaari et al. [126], all metals, save Pb, fell into the category of deficiency to minor enrichment, based on the geographic distributions of eight HMs (Al, Fe, Mn, Zn, Pb, Cu, Co, and Cd) in surface sediments of the northern Straits of Malacca.

ELTurk et al. [227] studied the distribution, enrichment, and ER of HMs in the Kuala Selangor estuary. The findings indicated that Pb levels in sediment were as high as the background value, implying anthropogenic contamination. ELTurk et al. [125] found that Pb concentrations in mangrove silt in the Klang estuary be above the threshold effect level, while Cu posed a medium environmental risk.

Hamzan et al. [122] studied the sediments from Kapar, Sungai Puloh, Sementa, and North Port. They found that the overall HM concentrations in each sediment layer fluctuated. Haris and Aris [119] also studied the intertidal surface sediments collected from Port Klang. In Port Klang, the enrichment factor and the geo-accumulation index revealed anthropogenic enrichments of Cd, Cu, Pb, and Zn. Cu concentrations had also surpassed the Canadian Interim Marine Sediment Quality Guideline at two locations near a commercial jetty, marina, and shipyard.

As the Selangor River (Kampung Kuantan) is bordered by residential and industrial sectors, as well as by particular leisure activities, agriculture, and fishing, the sediments in all plots and at all depths were contaminated with HMs, according to Nyangon et al. [127].

#### 3.8.2. East Coast of Peninsular Malaysia

Wang et al. [80] found that the surface sediments in the Kelantan River estuary and nearby shelf area were mostly clayey silt and that the total organic carbon content in sediments declined from the river to the shelf. Pb pollution was found in the surface sediments, while Cu and Zn levels showed unpolluted conditions. Natural weathering and erosion of rocks and soils in the watershed were thought to be the main sources of HM, which were enriched towards the river mouth.

Baruddin et al. [224] studied the bioavailability of sediment As, Cr, and Pb to the mangrove, *Rhizophora mucronata* in the Kelantan Delta, Malaysia. The majority of Pb (88.8%) was found in the residual geochemical fraction of the sediments. Based on samples collected from the Tanjung Lumpur mangrove forest, the computed enrichment factors (EF) found for Pb and Cu were judged to have anthropogenic inputs, according to Yunus et al. [222].

According to Shazili et al. [220], the HM levels were usually higher in the coastal sediments off Kuantan port (in the northern sector). This could be due to metal pollutants from land runoff through the Kuantan River estuary area, which was densely populated with oil and gas industries, a port, and urban neighborhoods. They discovered that the Northeast Monsoon affected the distribution of metals within the distinct HM fractions of sediment as well as shifting the sediments.

The enrichment factor along the Kelantan River was classified as highly considerable enrichment for Pb in nearshore areas, according to Pooveneswary et al. [82]. Ong et al. [78] found that the metal geochemistry of the surface sediments of Bidong Island (the South China Sea, East Coast of Peninsular Malaysia) was influenced by both natural and anthropogenic inputs to the watershed. Natural mechanisms were also shown to be more important than anthropogenic inputs in the concentration of metals, according to the researchers.

The influx of various rivers on the east coast of Peninsular Malaysia influences the nearshore areas there [126]. Weathering may cause those materials to degrade into a soluble form of Zn, which is then discharged into the aquatic environment [151]. Zn can be found in motor oil, grease, phosphate fertilizers, and sewage sludge, concrete, and undercoating [228].

The leachate from the Sabak beach waste area could also be a source of Cu in Kelantan waterways. The resulting leachate, which contained soluble Cu [229,230], could contaminate the coastal environment. These HMs could be carried downstream by the Kelantan River, which flows through densely populated urban areas like Tanah Merah and Kota Bharu.

#### 3.8.3. The Straits of Johore

Wood et al. [92] found that Pb and Zn levels in the Johor Straits were three and two times higher than world shale values, respectively, at sample sites along the Malaysia-Singapore causeway, which saw a lot of traffic. Pb enrichment was most likely caused by the use of leaded gasoline and Zn from tyre wear [92]. Many studies have been conducted on the Straits of Johore using the green-lipped mussel as a biomonitor of HM bioavailability. The results showed that the eastern part of the Johore Causeway in the Straits of Johore recorded higher metal levels [231,232,233,234,235,236]. Yap et al. [236] discovered higher HM levels of bioavailability and contamination by Cu and Zn in the eastern part of the Johore Straits (which is divided into two parts by a causeway) than in the western section, with Kg. Pasir Puteh in the eastern section has the highest bioavailability and contamination by HMs. These results agreed with those reported in the sediments [93].

#### 3.8.4. East Malaysia: Sabah and Sarawak

Tan et al. [225] studied the baseline HMs in the surface sediment of Marudu Bay. They showed that terrestrial organic materials dominated the C/N ratio of sediment in Marudu Bay. Except for Zn, which had greater concentrations in most sections of Marudu Bay, the HM values were equivalent to the baseline. In the riverine-mangrove environment of Kota Marudu, Aris et al. [85] assessed the quantity of metal buildup in sediment to identify possible pollution sources. Of the sediment cores from Marudu Bay (Kudat), Mamun et al. [217] found that the HM concentrations were mostly influenced by the weathering of sedimentary rocks and there was no significant influence by the weathered mafic and ultramafic rocks of the ophiolite sequence. Of the Mengkabong lagoon (Sabah), according to Praveena et al. [83], the Mengkabong mangrove sediments had background amounts of Cu and Zn and were unpolluted for Pb, based on the calculation of the geoaccumulation index.

Based on sample collection from beaches of Miri City (Sarawak), according to Nagarajan et al. [84], Cu and Zn levels were greater, indicating an external input rather than a natural process. Based on sediments collected from the Sadong river (Sarawak), Omorinoye et al. [218] reported that the EF values showed moderate to significant HM enrichment. Antonina et al. [212] examined surface sand samples from off the coasts of Sabah and Sarawak. They discovered that the values were higher, but that these were not necessarily due to human inputs, as high levels were also detected far from the coast. This could be due to particulate inflow from the Rajang River, as well as bottom sediment movement.

### 3.9. The Philippines

Six papers from the Philippines were not included in the calculations of ERI and HHRA [237,238,239,240,241,242]. The exclusions were mainly due to not all the three metals (Cu, Pb, and Zn) being analyzed and reported in the papers.

According to Japitana et al. [243], one of the water quality indicators that has to be importantly monitored is the amount of HMs, which can be detrimental to the aquatic environment of the Philippines and its human health when present at exceeding levels in water bodies classified as potable sources of water. Japitana et al. [243] aimed to identify the underlying challenges in monitoring HMs in the Philippines and to review the prospective tools and their integrations in the assessment, management, and rehabilitation of HMs that were most appropriate in the Philippine settings. The causes of HM contamination in Manila Bay ranged from natural abundance to pollution from non-point sources, such as home sewage, toxic industrial effluents from manufacturing and shipping activities, combustion emissions, mining operations, rubbish dump leachate, and runoff from chemical agriculture [133].

In a mine-tailing spill on the island of Marinduque, on 24 March 1996, David [132] found increased quantities (mg/kg dry weight) of Cu (706–3080), Pb (43–56), and Zn (131–276). Tailings sludge, which originated at the Marcopper Mine, poured down the Boac River abruptly and during later storm events.

Decena et al. [239] investigated HM contamination in the Mangonbangon River, a Tacloban City urban river. For Zn and Cu, the average enrichment factor indicated moderate and moderately severe enrichment, respectively; for Cu and Zn, the average contamination factor (Cf) indicated moderate contamination.

Elvira et al. [134] found that the HMs in the clay portions of the Laguna de Bay sediments had higher quantities. Based on a modified contamination rating, the sediments ranged from mildly contaminated to significantly contaminated. These findings demonstrated the need to take sediment compositions into account when assessing lake ecosystems. Of the Batan Bay in Aklan, Nillos et al. [240] reported the Cu was found to have the highest concentration (10.61–66.81 mg/kg dry weight) in the sediment. The Batan Bay sediments might be considered generally non-polluted concerning Cu, Pb, and Cd, Hence, these metals were not expected to cause adverse effects to aquatic organisms under normal conditions.

Prudente et al. [131] measured Fe, Mn, Zn, Cu, Pb, Ni, Cd, and Co contents in surface and core sediments from Manila Bay, as well as surface sediments from inflowing rivers. On the surface, core profiles indicated significantly variable and enhanced Cu, Pb, and Zn contents, indicating recent human inputs.

The need for the creation and execution of adequate regulatory measures for the protection and restoration of the HM-laden river in Zambales was highlighted by Sazon and Migo [242]. Based on sediments collected from Luzon Island, which included Laguna Lake and five of its tributary rivers, Vicente-Beckett [130] found that the average total Fe, Mn, Cu, Zn, Pb, Hg, and Cd contents suggested, in general, relatively low to moderate pollution.

## 4. Conclusions

As mentioned in the literature review, observations of HM contamination in the aquatic ecosystems of the ASEAN-5 nations had been undertaken using sediments for several years. Based on a review of the literature published from 1981 to February 2021, we found that the mean values of Cu, Pb, and Zn in the aquatic sediments of the ASEAN-5 countries were elevated and localized in high human activity sites in comparison to the earth’s upper continental crust and reference values for marine sediments. Based on 176 reports from 113 publications, the ranges of concentrations (mg/kg dry weight) were 0.09–3080 for Cu, 0.37–4950 for Zn, and 0.07–2666 for Pb. The ER values ranged from 0.02–1077 for Cu, 0.01–95.2 for Zn, and 0.02–784 for Pb. All reports (100%) showed the Zn ER values to be categorized as between ‘low potential ecological risk’ and ‘moderate potential ecological risk’. Almost all Cu ER values (98.3%) also showed similar ranges of the above two risk categories, except for a few reports. The highest Cu level (3080) was reported from a mine-tailing spill in Marinduque Island of the Philippines, with ‘very high ecological risk’. In addition, Semarang recorded Cu of ‘considerable potential ecological risk’ while polluted drainage sediments in the western part of Peninsular Malaysia were categorized as Cu ‘high potential ecological risk’. Almost all reports (96%) showed Pb ER values as being between ‘low potential ecological risk’ and ‘moderate potential ecological risk’ except for a few reports. A site in Semarang (Indonesia) showed Zn ER values to be ‘very high ecological risk’ (Pb level as 2666). Other reports that showed Pb values of ‘considerable potential ecological risk’ were one report (No. 24; Indonesia), two reports (No. 2 and 17; Thailand), two reports (No. 2 and 3; Vietnam), and one report (No. 21; west coast of Peninsular Malaysia). In terms of HHRA, all NCR values (HI values 1.0) for Cu, Pb, and Zn for ingestion and dermal contact routes for sediments from the ASEAN-5 countries exhibited no non-carcinogenic concern.

The current comparative ER and HHRA of Cu, Pb, and Zn would be valuable for the management and appropriate development of the area, as well as serve as a database for use in the future. The results of the study indicated that although the anthropogenic interventions to the aquatic ecosystems of the ASEAN-5 countries have not yet reached significant levels thus far regarding sediment contamination, it may be too late if swift and appropriate measures are not undertaken now for restricting future possible contamination.

This comprehensive evaluation is necessary to reveal the condition of the HM sink to surface sediment, allowing any changes in the concentration to be easily monitored and managed throughout the ASEAN-5 countries. It emphasizes the need of keeping the high diversity of mangroves to protect the coastal region’s health and productivity. The findings of HMs in sediments from studies conducted in the ASEAN-5 countries’ aquatic environments were highlighted in the review. The current review is significant not just in terms of aquatic animal health and management, but also provides a comparative basis for HMs. These findings could help raise awareness of the pollution-mitigation benefits that aquatic ecosystems naturally give, as well as provide data for future plans and regulations aimed at more sustainable development in these areas. Publications from the ASEAN-5 developing countries have risen fast too, possibly as a result of the growing concerns over severe HM pollution. The most common difficulties in the ASEAN-5 developing countries have been exposure route analyses and risk levels of public health noncarcinogenic concerns.

Human health hazards and impacts from HMs have become global concerns and researchers and managers are working to reduce human health risks caused by HM emissions and accumulation. We carried out this study from the perspectives of an historical literature review, research trends, and the growth of hot issues in the area. From this study, we found that the prevention and control of HMs’ human health concerns should be based on emission sources, exposure pathways, and receptor populations. Based on our review, we propose that the recent increasing trends warrant a need for further monitoring of HMs in the ASEAN-5 aquatic ecosystems.

### Recommendations

(1) Discharges from human activities and industry, which include processing plants, should be tightened up by upgraded regulatory standards. Regulation of anthropogenic sources should be prioritized to reduce HM emissions, improve mitigation and remediation measures in areas with high background concentrations, and minimize HM bioavailability and bioaccessibility to living organisms in various environmental compartments.
(2) For the management of marine protected areas in the ASEAN-5 countries, better cooperation of research activities among key governmental and non-governmental agencies should be continuous and promoted with investments and allocations of research fundings across the countries. Not just for environmental and health reasons, but also as a resource conservation measure, reprocessing or recycling of wastes containing HMs should be prioritized.
(3) In the future, it will be important to collect more HMs monitoring data to develop reliable inventories for not only the sediment but also for other environmental media in the ASEAN-5 countries. The intake of HM-contaminated commercial organisms that could potentially pose a significant risk to human health suggests that regular monitoring of HMs and emerging potentially toxic chemicals in the aquatic ecosystems should be highly advised.

## Figures and Tables

**Table 1:** Comparison of geographical areas, population, and gross domestic product (GDP) in the ASEAN-5 countries.

	Inland Area ^1^ (km^2^)	Water Area ^2^ (km^2^)	Population ^3^ (Million)	GDP 2010 ^4^ (Billions)	GDP 2021 ^4^ (Billions)	Capture Fisheries Output ^5^ (Thousand Tons)	Aquaculture Output ^5^ (Thousand Tons)
Indonesia	1,811,569	93,000	273.5	755.3	1167.2	6103	3849
Malaysia	329,613	1190	32.4	258.6	380.3	1489	261
Philippines	298,170	1830	109.6	208.4	398.3	2335	815
Thailand	510,890	2230	69.8	341.1	536.8	1844	1057
Vietnam	310,070	21,140	97.3	143.3	369.5	2804	3207
ASEAN-5	3,260,312	119,390	582.6	1706.6	2852.1	14,575	9189
World	148,940,000	361,132,000	7844.3	66,140.5	91,031.2	-	-

Note: ^1^ Data is taken from the United Nations Statistics Division [3]; ^2^ Water area: the sum of the surface areas of all inland water bodies (lakes, reservoirs, and rivers) within international boundaries and coastlines. Coastal internal waters (some small bays) may be included [4]; ^3^ Worldometer [2]; ^4^ World Economic Outlook Database (WEO, October 2020). ^5^ Aquaculture and capture fisheries output among ASEAN members and top producer countries worldwide, 2013 [5].

**Table 2:** Concentrations (mg/kg dry weight) of Cu, Pb, and Zn reported from the ASEAN-5 countries obtained from the literature from 1981–2021.

Country	No.		Locations	Cu	Zn	Pb	References
Indonesia (N = 15)	1	min	Coast of Pantai Ujung Watu (Central Java)	2.10	0.60	1.10	Razak [41] ^nS^
	2	max	Coast of Pantai Ujung Watu (Central Java)	29.6	9.30	10.9	Razak [41] ^nS^
	3	mean	Jakarta Bay, Indonesia	82.9	79.8	438	Soegiarto [42] ^nS^
	4	min	Estuaries of Brantas River and Solo River	24.0	40.0	9.00	Everaarts [18] ^S^
	5	max	Estuaries of Brantas River and Solo River	58.0	122	23.0	Everaarts [18] ^S^
	6	min	Semarang, Central Java	12.3	53.7	5.20	Widianarko et al. [43] ^S^
	7	max	Semarang, Central Java	448	1257	2666	Widianarko et al. [43] ^S^
	8	min	Jakarta Bay	13.0	68.0	17.0	Williams et al. [44] ^S^
	9	max	Jakarta Bay	25.0	95.0	28.0	Williams et al. [44] ^S^
	10	mean	Telaga Tujuh Karimun, Riau Achipelago	46.3	96.8	88.2	Amin [45] ^nS^
	11	mean	Berau Delta	16.5	6.10	12.2	Arifin [46] ^nS^
	12	min	Dumai coast	1.61	31.5	14.6	Amin et al. [19] ^S^
	13	max	Dumai coast	13.8	87.1	84.9	Amin et al. [19] ^S^
	14	min	Jakarta Bay 2010	10.8	85.0	13.0	Riyadi et al. [47] ^nS^
	15	max	Jakarta Bay 2010	107	845	106	Riyadi et al. [47] ^nS^
	16	min	Jakarta Bay 2004	0.82	53.9	3.64	Rochyatun and Rozak [48] ^nS^
	17	max	Jakarta Bay 2004	74.7	498	53.0	Rochyatun and Rozak [48] ^nS^
	18	mean	Jakarta Bay	10.0	33.0	9.00	Hosono et al. [49] ^S^
	19	mean	Mahakam Delta, East Kalimantan	27.7	187	27.6	Effendi et al. [17] ^S^
	20	min	river and coast sediments of the Jakarta Bay	14.8	64.2	3.00	Sindern et al. [50] ^S^
	21	max	river and coast sediments of the Jakarta Bay	355	1130	210	Sindern et al. [50] ^S^
NonS	22	min	Tanjung Jati power plant in Jepara District, Central Java	6.98	271	0.33	Isworo and Oetari [51] ^nS^
NonS	23	max	Tanjung Jati power plant in Jepara District, Central Java	11.0	681	3.88	Isworo and Oetari [51] ^nS^
	24	mean	North-West Coast of Kundur Island, Kepulauan Riau Province	1.70	11.5	292	Amin et al. [52] ^S^
Thailand (N = 11)	1	min	The Upper Gulf of Thailand	3.00	35.0	20.0	Menasveta and Cheevaparanapiwat [53] ^S^
	2	max	The Upper Gulf of Thailand	37.0	95.0	283	Menasveta and Cheevaparanapiwat [53] ^S^
	3	max	Ao Ban Don and Pattani Bay	70.0	250	424	Everaarts and Swennen [54] ^nS^
	4	mean	River near Uthai District (Phra Nakhon Si Ayutthaya Province)	20.0	60.0	22.0	Mingkhwan and Worakhunpiset [55] ^S^
	5	mean	River Bangpa-in District (Phra Nakhon Si Ayutthaya Province)	100	70.0	16.0	Mingkhwan and Worakhunpiset [55] ^S^
	6	min	Pattany Bay	22.5	75.0	79.4	Everaarts et al. [56] ^nS^
	7	max	Pattany Bay	26.90	90.0	97.0	Everaarts et al. [56] ^nS^
	8	min	Upper Gulf of Thailand, rivers of Mae Klong, Tha Chin, Chao Phraya, and Bang Pakong	6.00	18.0	13.0	Qiao et al. [57] ^S^
	9	max	Upper Gulf of Thailand, rivers of Mae Klong, Tha Chin, Chao Phraya, and Bang Pakong	36.0	127	36.0	Qiao et al. [57] ^S^
	10	mean	Chanthaburi Province	7.41	18.1	1.82	Potipat et al. [58] ^S^
	11	mean	Pattani Bay	3.27	9.33	9.57	Pradit et al. [59] ^S^
	12	min	Western Gulf of Thailand	2.20	1.63	4.13	Liu et al. [60] ^S^
	13	max	Western Gulf of Thailand	25.3	78.9	38.7	Liu et al. [60] ^S^
	14	min	mangrove forest stems from Surat Thani Province	2.90	7.50	8.15	Pumijumnong and Danpradit [61] ^S^
	15	max	mangrove forest stems from Surat Thani Province	13.7	46.4	26.0	Pumijumnong and Danpradit [61] ^S^
	16	min	Mangrove Pattani Bay	3.30	30.4	166	Kaewtubtim et al. [13] ^S^
	17	max	Mangrove Pattani Bay	18.7	39.8	314	Kaewtubtim et al. [13] ^S^
	18	mean	Chao Phraya River in Bangkok	214	240	62.6	Wijaya et al. [62] ^S^
Vietnam (N = 15)	1	min	Rivers in Hanoi	37.0	93.0	43.0	Ho and Egashira [63] ^nS^
	2	max	Rivers in Hanoi	309	4950	361	Ho and Egashira [63] ^nS^
	3	mean	Paddy field near Red River Delta	193	381	340	Phuong et al. [28] ^S^
	4	mean	Cua Ong Habor, Ha Long Bay	20.0	40.0	16.0	Ho et al. [64] ^S^
	5	min	Lang Co, Truong Giang, An Khe, Nuoc Man, Nuoc Ngot, Thi Nai, O Loan, Thuy Trieu and Dam Nai	6.58	31.0	17.4	Romano et al. [15] ^nS^
	6	max	Lang Co, Truong Giang, An Khe, Nuoc Man, Nuoc Ngot, Thi Nai, O Loan, Thuy Trieu and Dam Nai	28.6	92.0	33.8	Romano et al. [15] ^nS^
	7	min	Cua Ong, Ha Long	13.0	12.0	4.00	Tue et al. [65] ^S^
	8	max	Cua Ong, Ha Long	30.0	94.0	41.0	Tue et al. [65] ^S^
	9	mean	The Cam River mouth (Haiphong Province), Red River System	82.0	178	92.0	Ho et al. [66] ^S^
	10	mean	Cam River-Mouth, Haiphong	14.0	65.0	18.0	Ho et al. [67] ^S^
	11	mean	Ninh Hoa district central of Vietnam	12.0	79.0	34.0	Dung et al. [68] ^S^
	12	min	Cai River estuary and Nha Trang Bay	18.1	63.8	33.0	Koukina et al. [16] ^S^
	13	max	Cai River estuary and Nha Trang Bay	73.8	118	86.2	Koukina et al. [16] ^S^
	14	min	Red River	20.0	40.0	27.0	Nguyen et al. [69] ^S^
	15	max	Red River	332	287	188	Nguyen et al. [69] ^S^
	16	min	Thi Vai Estuary and in the Can Gio Mangrove Forest	16.5	73.0	17.0	Costa-Böddeker et al. [25] ^S^
	17	max	Thi Vai Estuary and in the Can Gio Mangrove Forest	48.5	127	25.0	Costa-Böddeker et al. [25] ^S^
	18	mean	mangrove forest sediment in Ho Chi Minh City	26.0	113	26.0	Dung et al. [26] ^S^
	19	min	Tidal flats in the north of Vietnam from Quang Ninh to Ninh Binh provinces	0.69	3.95	5.78	Hoai et al. [70] ^S^
	20	max	Tidal flats in the north of Vietnam from Quang Ninh to Ninh Binh provinces	94.8	492	120	Hoai et al. [70] ^S^
	21	min	Ha Long Bay	3.80	6.34	10.2	Dang Hoai et al. [71] ^S^
	22	max	Ha Long Bay	41.7	119	69.9	Dang Hoai et al. [71] ^S^
	23	min	Duyen Hai Seaport Area in Tra Vinh Province	3.07	82.9	65.3	Tham et al. [72] ^S^
	24	max	Duyen Hai Seaport Area in Tra Vinh Province	9.15	212	85.2	Tham et al. [72] ^S^
East PM (N = 12)	1	mean	Paka Estuary, Terengganu	29.2	72.5	54.9	Kamaruzzaman et al. [73] ^S^
	2	mean	Mangrove forests of Terengganu	31.1	20.8	10.5	Kamaruzzaman et al. [73] ^nS^
	3	mean	Kerteh mangrove Forest, Terengganu	29.0	22.3	11.7	Kamaruzzaman and Ong [74] ^S^
	4	mean	Kelantan River, Kelantan	6.74	18.7	20.8	Dasar et al. [75] ^S^
	5	mean	East coast PM, South China Sea	37.4	9.30	44.3	Rezaee Ebrahim Saraee et al. [76] ^S^
	6	mean	East coast of Pahang	0.69	0.75	0.29	Redzwan et al. [77] ^S^
	7	mean	Bidong Island, South China sea Cu	19.3	61.3	20.5	Ong et al. [78] ^nS^
	8	mean	Balok River, Pahang	24.3	159	29.3	Mohd Zahari abdullah et al. [79] ^S^
	9	mean	Setiu Wetlands, Terengganu	0.38	2.15	1.60	Pradit et al. [59] ^S^
	10	min	Kelantan River, Kelantan	16.0	47.6	42.2	Wang et al. [80] ^S^
	11	max	Kelantan River, Kelantan	25.2	63.5	65.5	Wang et al. [80] ^S^
	12	max	Terengganu coast, Terengganu	36.6	41.1	19.8	Mohamed and Shazili [81] ^S^
	13	mean	Kelantan riverine, Kelantan	11.4	32.9	22.6	Pooveneswary et al. [82] ^S^
	14	mean	Kelantan nearshore area, Kelantan	14.1	41.0	62.2	Pooveneswary et al. [82] ^S^
East Malaysia (N = 8)	1	min	Mengkabong lagoon, Sabah	19.0	41.0	41.0	Praveena et al. [83] ^S^
	2	max	Mengkabong lagoon, Sabah	28.0	57.0	52.0	Praveena et al. [83] ^S^
	3	min	Forty-three sediment samples were collected from the beaches of Miri City, Sarawak	28.8	17.9	10.5	Nagarajan et al. [84] ^S^
	4	max	Forty-three sediment samples were collected from the beaches of Miri City, Sarawak	71.3	28.3	19.7	Nagarajan et al. [84] ^S^
	5	mean	Kota Marudu, Sabah	248	451	53.6	Aris et al. [85] ^S^
	6	mean	Mamut River, Sabah	583	61.4	23.5	Muhammad Ali et al. [86] ^S^
	7	mean	Tuaran, Sabah	6.49	22.0	2.63	Tan et al. [87] ^nS^
	8	min	Liwagu River and Mansahaban River at Ranau Sabah	8.07	16.0	4.45	Tair and Eduin [88] ^nS^
	9	max	Liwagu River and Mansahaban River at Ranau Sabah	391	69.3	32.60	Tair and Eduin [88] ^nS^
	10	mean	Baleh River, Sarawak	19.9	55.0	7.55	Chai et al. [89] ^nS^
	11	max	Sadong River, Sarawak	0.09	6.27	0.07	Asare et al. [90] ^nS^
Straits of Johore (N = 4)	1	mean	Straits of Johore, Johore	4.40	26.1	30.8	Mat et al. [91] ^nS^
	2	min	Strait of Johor, Johore	10.8	68.5	26.4	Wood et al. [92] ^S^
	3	max	Strait of Johor, Johore	92.9	231	69.9	Wood et al., [92] ^S^
	4	mean	Strait of Johor, Johore	57.8	210	52.5	Zulkifli et al. [10] ^S^
	5	mean	Eastern part of the Straits of Johore (3 sites; 2004)	110	180	33.6	Yap et al. [93] ^S^
	6	mean	The western part of the Straits of Johore (7 sites; 2004)	28.6	137	33.7	Yap et al. [93] ^S^
West PM (N = 41)	1	min	Penang Island	1.00	8.00	6.00	Sivalingam et al. [94] ^nS^
	2	max	Penang Island	26.0	76.0	35.0	Sivalingam et al. [94] ^nS^
	3	mean	Jeram coast, Perak	40.0	100	48.0	Everaarts and Swennen [54] ^nS^
	4	min	Juru, Prai Industrial Estate, Penang	9.30	73.5	20.8	Seng et al. [95] ^S^
	5	max	Juru, Prai Industrial Estate, Penang	13.8	110	33.0	Seng et al. [95] ^S^
	6	max	West coast of Peninsular Malaysia	9.99	1400	45.0	Ismail et al. [96] ^S^
	7	min	Bintulu coast	7.00	39.0	11.0	Ismail [96] ^S^
	8	max	Bintulu coast	13.0	91.0	36.0	Ismail [96] ^S^
	9	min	Juru River, Penang	2.00	2.00	0.10	Lim and Kiu [97] ^S^
	10	max	Juru River, Penang	144	483	117	Lim and Kiu [97] ^S^
	11	mean	Sepang Besar River, Selangor	23.0	55.0	16.0	Ismail and Ramli [7] ^S^
	12	min	Prai, Industrial Zone, Penang	9.99	30.3	22.2	Ismail and Asmah [98] ^nS^
	13	max	Prai, Industrial Zone, Penang	63.4	513	45.3	Ismail and Asmah [98] ^nS^
	14	min	Offshore area of the west coast of Peninsular Malaysia 1998–1999 (20 sites)	0.25	4.00	3.59	Yap et al. [99,100] ^S^
	15	max	Offshore area of the west coast of Peninsular Malaysia 1998–1999 (20 sites)	13.8	79.1	25.4	Yap et al. [99,100] ^S^
	16	min	Intertidal area of the west coast of Peninsular Malaysia 1999–2001 (26 sites)	0.40	3.12	0.96	Yap et al. [99,100] ^S^
	17	max	Intertidal area of the west coast of Peninsular Malaysia 1999–2001 (26 sites)	315	306	69.8	Yap et al. [99,100] ^S^
	18	min	Southwestern coast of Peninsular Malaysia 2005 (7 sites)	3.43	43.1	16.5	Yap et al. [101] ^nS^
	19	max	Southwestern coast of Peninsular Malaysia 2005 (7 sites)	154	324	67.8	Yap et al. [101] ^nS^
	20	min	Sri Serdang Industrial area drainage, Selangor	43.0	169	56.1	Yap et al. [102] ^nS^
	21	max	Sri Serdang Industrial area drainage, Selangor	551	296	404	Yap et al. [102] ^nS^
	22	min	Polluted drainage sediments in the west part of Peninsular Malaysia (6 sites)	877	330	57.4	Yap et al. [103] ^nS^
	23	max	Polluted drainage sediments in the west part of Peninsular Malaysia (6 sites)	1019	484	1267	Yap et al. [103] ^nS^
	24	min	Six intertidal areas and four urban drainage sites, Selangor	6.64	50.2	26.0	Yap et al. [104,105] ^S^
	25	max	Six intertidal areas and four urban drainage sites, Selangor	123	336	228	Yap et al. [104,105] ^S^
	26	mean	Juru Estuary, Penang	32.9	317	30.2	Yap and Tan [106] ^nS^
	27	mean	Beach sediment in Langkawi Island, Kedah	6.00	34.7	63.7	Razi-Idris et al. [107] ^S^
	28	mean	Juru River, Penang	32.0	531	60.8	Yap et al. [108] ^nS^
	29	min	Klang River Estuary, Selangor	5.29	46.4	30.4	Yap et al. [109] ^S^
	30	max	Klang River Estuary, Selangor	53.9	207	62.3	Yap et al. [109] ^S^
	31	min	Langat River, Selangor	5.20	46.9	3.06	Yap and Mohd Khairul [110] ^nS^
	32	max	Langat River, Selangor	12.3	77.7	8.17	Yap and Mohd Khairul [110] ^nS^
	33	min	West Port, Selangor	7.40	23.0	22.3	Sany et al. [111] ^nS^
	34	max	West Port, Selangor	27.6	98.3	80.0	Sany et al. [111] ^nS^
	35	min	Northern coastal sediments of Peninsular Malaysia (13 sites, 2005)	4.79	33.6	15.9	Yap and Pang [31,32] ^S^
	36	max	Northern coastal sediments of Peninsular Malaysia (13 sites, 2005)	32.9	317	61.6	Yap and Pang [31,32] ^S^
	37	min	Northern drainage sediment of Peninsular Malaysia (5 sites, 2005)	10.2	88.7	10.2	Yap and Pang [31,32] ^S^
	38	max	Northern drainage sediment of Peninsular Malaysia (5 sites, 2005)	120	484	126	Yap and Pang [31,32] ^S^
	39	mean	Sg. Buloh, Selangor	34.7	94.0	37.3	Nemati et al. [112] ^S^
	40	max	Upstream Perak River, Perak	19.1	51.3	27.0	Salam et al. [113] ^S^
	41	max	Downstream Perak River, Perak	60.0	161	60.8	Salam et al. [113] ^S^
	42	max	Sungai Puloh mangrove, Klang, Selangor	202	651	225	Abubakar et al. [114] ^S^
	43	max	Juru, Penang	43.0	83.7	35.5	Wood et al. [115] ^nS^
	44	mean	Langat River, Selangor	5.72	35.9	30.4	Lim et al. [116] ^S^
	45	mean	Klang Straits (West Port, North Port, and South Port), Selangor	17.4	51.1	59.5	Sany et al. [8,29] ^S^
	46	min	Perlis River, Perlis	7.31	61.3	23.5	Jamil et al. [117] ^nS^
	47	max	Perlis River, Perlis	35.9	121	76.7	Jamil et al. [117] ^nS^
	48	mean	Langat River, Selangor	2.68	29.7	15.5	Shafie et al. [118] ^S^
	49	mean	West coast of Negeri Sembilan and Melaka	0.18	0.37	0.56	Redzwan et al. [77] ^S^
	50	mean	Port Klang, Selangor	6.80	48.1	15.6	Haris and Aris [119] ^S^
	51	mean	Sungai Puloh mangrove, Klang, Selangor	46.9	1024	78.8	Udechukwu et al. [9] ^S^
	52	max	Klang River, Selangor	52.9	272	64.1	Naji and Ismail [120] ^S^
	53	min	West coast mangrove of Peninsular Malaysia 2011 (9 sites)	5.59	29.4	25.4	Cheng and Yap [121] ^S^
	54	max	West coast mangrove of Peninsular Malaysia 2011 (9 sites)	28.7	130	173	Cheng and Yap [121] ^S^
	55	min	Klang coastal area (Kapar, Sungai Puloh, Sementa and North Port), Selangor	3.20	73.2	8.00	Hamzan et al. [122] ^S^
	56	max	Klang Coastal area (Kapar, Sungai Puloh, Sementa and North Port), Selangor	10.2	125	25.7	Hamzan et al. [122] ^S^
	57	min	Bayan Lepas Free Industrial Zone of Penang	1.92	8.86	7.74	Khodami et al. [123] ^S^
	58	max	Bayan Lepas Free Industrial Zone of Penang	387	103	63.2	Khodami et al. [123] ^S^
	59	min	Langat River, Selangor	10.5	55.6	48.1	Wong et al. [124] ^S^
	60	max	Langat River, Selangor	17.4	84.7	64.3	Wong et al. [124] ^S^
	61	mean	Kuala Selangor estuary, Selangor	3.55	76.6	28.8	ELTurk et al. [125] ^S^
	62	min	Northern Malacca Strait	3.50	30.1	8.88	Shaari et al. [126] ^S^
	63	max	Northern Malacca Strait	16.6	79.4	29.3	Shaari et al. [126] ^S^
	64	min	Mangrove sediments along the Selangor River, Selangor	1.00	215	8.83	Nyangon et al. [127] ^S^
	65	max	Mangrove sediments along the Selangor River, Selangor	10.6	259	28.6	Nyangon et al. [127] ^S^
	66	mean	Mangrove sediment in the Klang estuary, Selangor	9.38	22.5	37.40	ELTurk et al. [125] ^S^
	67	min	Juru estuary, Penang	9.56	72.1	15.1	Yusoff et al. [128] ^S^
	68	max	Juru estuary, Penang	64.9	304	59.5	Yusoff et al. [128] ^S^
Philippines (N = 7)	1	mean	Laguna Lake(Laguna de Bay)	100	114	10.4	Vicente-Beckett [129,130] ^S^
	2	min	Manila Bay	32.0	60.0	6.00	Prudente et al. [131] ^S^
	3	max	Manila Bay	118	329	95.0	Prudente et al. [131] ^S^
	4	min	a mine-tailings spill, Marinduque Island	706	13.0	43.0	David [132] ^S^
	5	max	a mine-tailings spill, Marinduque Island	3080	276	56.0	David [132] ^S^
	6	min	Manila Bay (core sediments)	22.9	50.0	7.30	Hosono et al. [14] ^S^
	7	max	Manila Bay (core sediments)	38.6	96.0	19.0	Hosono et al. [14] ^S^
	8	min	Manila Bay (core sediments)	56.7	74.6	8.69	Olivares et al. [133] ^S^
	9	max	Manila Bay (core sediments)	90.3	122	26.6	Olivares et al. [133] ^S^
	10	min	Laguna de Bay	66.8	112	20.4	Elvira et al. [134] ^S^
	11	max	Laguna de Bay	147	251	64.9	Elvira et al. [134] ^S^

Note: PM = Peninsular Malaysia. ^S^ = Scopus; ^nS^ = non-Scopus.

**Table 3:** Reference values and exposure factors were employed to estimate the intake values and health risks of heavy metals in sediments for the present study.

Factor	Definition	Unit	Children	Adults	References
ABF	Dermal absorption factor	unitless	0.001	0.001	Chabukdhara and Nema [140]
AF	Skin adherence factor	mg/cm day	0.2	0.7	US EPA [142]
AT	Average time	days	365 × ED	365 × ED	US EPA [137]
BW	Body weight of the exposed individual	kg	15	55.9	Beijing Quality and Technology Supervision Bureau [143]
ED	Exposure duration	years	6	24	USEPA [139]
EF	Exposure frequency	days/year	350	350	Beijing Quality and Technology Supervision Bureau [143]
IngR	Ingestion rate of soil	mg/day	200	100	USEPA [139]
PEF	Particle emission factor	m^3^/kg	1.36 × 10^9^	1.36 × 10^9^	USEPA [139]
SA	Exposed skin surface area	cm^2^	1600	4350	Beijing Quality and Technology Supervision Bureau (2009)

**Table 4:** Overall statistics of values of concentrations (mg/kg dry weight) of Cu, Zn, and Pb, and their concentration factors (Cf) and ecological risks (ER) from the present study.

Indonesia (N = 24)	Cu	Zn	Pb	Cu Cf	Zn Cf	Pb Cf	Cu ER	Zn ER	Pb ER	PERI
Minimum	0.82	0.60	0.33	0.06	0.01	0.02	0.29	0.01	0.10	1.07
Maximum	448	1257	2666	31.3	24.2	157	157	24.2	784	965
Mean	58.1	242	172	4.06	4.65	10.1	20.3	4.65	50.5	75.4
Median	15.7	82.4	15.8	1.09	1.58	0.93	5.47	1.58	4.65	15.9
SD	110	364	541	7.71	7.01	31.9	38.5	7.01	159	196
SE	22.5	74.4	110	1.57	1.43	6.51	7.87	1.43	32.53	40.1
Skewness	2.78	1.78	4.32	2.78	1.78	4.32	2.78	1.78	4.32	4.12
Kurtosis	6.52	1.83	17.4	6.52	1.83	17.4	6.52	1.83	17.4	16.2
Thailand (N = 18)	Cu	Zn	Pb	Cu Cf	Zn Cf	Pb Cf	Cu ER	Zn ER	Pb ER	PERI
Minimum	2.20	1.63	1.82	0.15	0.03	0.11	0.77	0.03	0.54	2.02
Maximum	214	250	424	15.0	4.81	24.9	74.8	4.81	125	154
Mean	34.0	71.8	90.1	2.38	1.38	5.30	11.9	1.38	26.5	39.8
Median	19.4	53.2	31.0	1.36	1.02	1.82	6.76	1.02	9.12	23.7
SD	51.7	71.8	125	3.62	1.38	7.34	18.1	1.38	36.7	44.0
SE	12.2	16.9	29.4	0.85	0.33	1.73	4.26	0.33	8.65	10.4
Skewness	2.61	1.52	1.61	2.61	1.52	1.61	2.61	1.52	1.61	1.27
Kurtosis	6.36	1.45	1.30	6.37	1.45	1.30	6.36	1.45	1.30	0.60
Vietnam (N = 24)	Cu	Zn	Pb	Cu Cf	Zn Cf	Pb Cf	Cu ER	Zn ER	Pb ER	PERI
Minimum	0.69	3.95	4.00	0.05	0.08	0.24	0.24	0.08	1.18	2.02
Maximum	332	4950	361	23.2	95.2	21.2	116	95.2	106	309
Mean	59.7	323	73.3	4.18	6.21	4.31	20.9	6.21	21.6	48.7
Median	23.0	92.5	33.9	1.61	1.78	2.00	8.04	1.78	9.97	21.8
SD	90.5	992	95.4	6.33	19.1	5.61	31.7	19.1	28.1	72.3
SE	18.5	203	19.5	1.29	3.90	1.15	6.46	3.90	5.73	14.8
Skewness	2.19	4.49	2.16	2.19	4.49	2.16	2.19	4.49	2.16	2.49
Kurtosis	3.59	18.4	3.69	3.59	18.4	3.69	3.59	18.4	3.69	5.57
Malaysia (N = 99)	Cu	Zn	Pb	Cu Cf	Zn Cf	Pb Cf	Cu ER	Zn ER	Pb ER	PERI
Minimum	0.09	0.37	0.07	0.01	0.01	0.00	0.03	0.01	0.02	0.17
Maximum	1019	1400	1267	71.3	26.9	74.5	356	26.9	373	738
Mean	70.0	145	55.7	4.89	2.78	3.28	24.47	2.78	16.4	43.6
Median	17.4	69.3	30.4	1.22	1.33	1.79	6.09	1.33	8.94	17.1
SD	164	210	134	11.4	4.04	7.89	57.2	4.04	39.5	90.2
SE	16.4	21.1	13.5	1.15	0.41	0.79	5.75	0.41	3.97	9.06
Skewness	3.99	3.33	7.76	3.99	3.33	7.76	3.99	3.33	7.76	5.41
Kurtosis	16.9	14.2	66.0	16.9	14.2	66.0	16.9	14.2	66.0	35.4
Philippines (N = 11)	Cu	Zn	Pb	Cu Cf	Zn Cf	Pb Cf	Cu ER	Zn ER	Pb ER	PERI
Minimum	22.9	50.0	6.00	1.60	0.96	0.35	8.01	0.96	1.76	11.1
Maximum	3080	329	95.0	215	6.33	5.59	1077	6.33	27.9	1099
Mean	405	147	32.5	28.3	2.82	1.91	142	2.82	9.55	154
Median	90.3	114	20.4	6.31	2.19	1.20	31.57	2.19	6.00	40.2
SD	908	94.1	28.9	63.5	1.81	1.70	317	1.81	8.51	321
SE	274	28.4	8.73	19.1	0.55	0.51	95.7	0.55	2.57	96.8
Skewness	2.64	0.90	0.99	2.64	0.90	0.99	2.64	0.90	0.99	2.62
Kurtosis	5.31	−0.65	−0.14	5.31	−0.65	−0.14	5.31	−0.65	−0.14	5.25
ASEAN-5 (N = 176)	Cu	Zn	Pb	Cu Cf	Zn Cf	Pb Cf	Cu ER	Zn ER	Pb ER	PERI
Minimum	0.09	0.37	0.07	0.01	0.01	0.00	0.03	0.01	0.02	0.17
Maximum	3080	4950	2666	215	95.2	157	1077	95.2	784	1099
Mean	84.2	175	76.0	5.89	3.37	4.47	29.5	3.37	22.4	55.2
Median	20.0	74.8	29.0	1.40	1.44	1.71	6.99	1.44	8.54	19.5
SD	268	422	230	18.8	8.11	13.6	93.9	8.11	67.8	131
SE	20.2	31.8	17.4	1.41	0.61	1.02	7.07	0.61	5.11	9.88
Skewness	8.53	8.70	9.03	8.53	8.70	9.03	8.53	8.70	9.03	5.81
Kurtosis	87.8	92.3	93.1	87.81	92.3	93.1	87.8	92.3	93.1	38.1

Note: PM = Peninsular Malaysia.

**Table 5:** Overall statistics of values of hazard quotient ingestion (HQ_ing_), hazard quotient dermal (HQ_dermal_), and hazard index (HI) of Cu, Pb, and Zn for children and adults from ASEAN-5 (N = 176) were obtained in the present study.

Cu	Children HQ_ing_	Children HQ_dermal_	Children HI	Adult HQ_ing_	Adult HQ_dermal_	Adult HI
Minimum	2.95 × 10^−5^	1.57 × 10^−7^	2.97 × 10^−5^	3.96 × 10^−6^	4.02 × 10^−7^	4.36 × 10^−6^
Maximum	**1.01**	5.38 × 10^−3^	1.01	1.35 × 10^−1^	1.37 × 10^−2^	1.49 × 10^−1^
Mean	2.76 × 10^−2^	1.47 × 10^−4^	2.77 × 10^−2^	3.70 × 10^−3^	3.76 × 10^−4^	4.08 × 10^−3^
Median	6.55 × 10^−3^	3.50 × 10^−5^	6.59 × 10^−3^	8.79 × 10^−4^	8.93 × 10^−5^	9.69 × 10^−4^
SD	8.80 × 10^−2^	4.69 × 10^−4^	8.81 × 10^−2^	1.18 × 10^−2^	1.19 × 10^−3^	1.30 × 10^−2^
SE	6.63 × 10^−3^	3.53 × 10^−5^	6.64 × 10^−3^	8.88 × 10^−4^	9.01 × 10^−5^	9.79 × 10^−4^
Skewness	8.54	8.54	8.50	8.51	8.52	8.52
Kurtosis	8.79 × 10	8.78 × 10	8.73 × 10	8.75 × 10	8.75 × 10	8.76 × 10
Pb	Children HQ_ing_	Children HQ_dermal_	Children HI	Adult HQ_ing_	Adult HQ_dermal_	Adult HI
Minimum	2.59 × 10^−4^	2.80 × 10^−6^	2.62 × 10^−4^	3.48 × 10^−5^	7.14 × 10^−6^	4.19 × 10^−5^
Maximum	**9.87**	1.07 × 10^−1^	9.98	1.32	2.72 × 10^−1^	1.60
Mean	2.81 × 10^−1^	3.04 × 10^−3^	2.84 × 10^−1^	3.77 × 10^−2^	7.75 × 10^−3^	4.55 × 10^−2^
Median	1.08 × 10^−1^	1.16 × 10^−3^	1.09 × 10^−1^	1.44 × 10^−2^	2.97 × 10^−3^	1.74 × 10^−2^
SD	8.53 × 10^−1^	9.23 × 10^−3^	8.62 × 10^−1^	1.14 × 10^−1^	2.35 × 10^−2^	1.38 × 10^−1^
SE	6.43 × 10^−2^	6.96 × 10^−4^	6.50 × 10^−2^	8.60E-03	1.77 × 10^−3^	1.04 × 10^−2^
Skewness	9.03	9.05	9.03	9.01	9.03	9.04
Kurtosis	9.31 × 10	9.36 × 10	9.31 × 10	9.28 × 10	9.32 × 10	9.33 × 10
Zn	Children HQ_ing_	Children HQ_dermal_	Children HI	Adult HQ_ing_	Adult HQ_dermal_	Adult HI
Minimum	1.62 × 10^−5^	1.29 × 10^−7^	1.63 × 10^−5^	2.17 × 10^−6^	3.30 × 10^−7^	2.50 × 10^−6^
Maximum	2.16 × 10^−1^	1.73 × 10^−3^	2.18 × 10^−1^	2.90 × 10^−2^	4.42 × 10^−3^	3.34 × 10^−2^
Mean	7.65 × 10^−3^	6.12E × 10^−5^	7.71 × 10^−3^	1.03 × 10^−3^	1.56 × 10^−4^	1.18 × 10^−3^
Median	3.27 × 10^−3^	2.62 × 10^−5^	3.29 × 10^−3^	4.39 × 10^−4^	6.67 × 10^−5^	5.06 × 10^−4^
SD	1.84 × 10^−2^	1.47 × 10^−4^	1.86 × 10^−2^	2.47 × 10^−3^	3.77 × 10^−4^	2.85 × 10^−3^
SE	1.39 × 10^−3^	1.11 × 10^−5^	1.40 × 10^−3^	1.86 × 10^−4^	2.84 × 10^−5^	2.15 × 10^−4^
Skewness	8.70	8.71	8.70	8.70	8.71	8.70
Kurtosis	9.22 × 10	9.24 × 10	9.23 × 10	9.23 × 10	9.24 × 10	9.22 × 10

Note: Values in bold indicate HQ values > 1.0; PM = Peninsular Malaysia.

## Data Availability

Not applicable.

## Supplementary Materials

The following are available online at https://www.mdpi.com/article/10.3390/biology11010007/s1, Table S1: Concentrations (mg/kg dry weight) of Cu, Pb and Zn based on Sediment Quality Guidelines and reference values, Table S2: Values of concentration factors (Cf), ecological risk (ER), and potential ecological risk index (PERI) calculated from the present study based on the cited concentrations of Cu, Pb and Zn reported from ASEAN-5 countries, Table S3: Values of hazard quotient ingestion (HQ_ing_), hazard quotient dermal (HQ_dermal_) and hazard index (HI) of Cu for children and adults from the present study, Table S4: Values of hazard quotient ingestion (HQ_ing_), hazard quotient dermal (HQ_dermal_) and hazard index (HI) of Pb for children and adults from the present study, Table S5: Values of hazard quotient ingestion (HQ_ing_), hazard quotient dermal (HQ_dermal_) and hazard index (HI) of Zn for children and adults from the present study.
