# Supplementary material for: Ecological-Health Risk Assessments of Heavy Metals (Cu, Pb, and Zn) in Aquatic Sediments from the ASEAN-5 Emerging Developing Countries: A Review and Synthesis
Source: Biology (Basel). 2021 Dec 21;11(1):7. doi: 10.3390/biology11010007 (PMC8773003; doi:10.3390/biology11010007)
Supplement: Supplementary file 1 [file biology-11-00007-s001.zip › biology-1515388-supplementary.pdf]

Supplementary Materials

Ecological-health risk assessments of heavy metals (Cu, Pb and Zn) in aquatic sediments from the ASEAN-5 emerging developing countries: A review and synthesis

Chee Kong Yap[1]* and Khalid Awadh Al-Mutairi[2]

[1]Department of Biology, Faculty of Science, Universiti Putra Malaysia, 43400 UPM, Serdang, Selangor, Malaysia
[2]Department of Biology, Faculty of Science, University of Tabuk, Tabuk, P.O. Box 741, Saudi Arabia
*Corresponding author: yapckong@hotmail.com; yapchee@upm.edu.my

Table S1: Concentrations (mg/kg dry weight) of Cu, Pb and Zn based on Sediment Quality Guidelines and reference values.

Sediment Quality Guidelines	Cu	Zn	Pb	References
ISQV-low	65.00	200.00	75.00	Chapman et al. (1999)
ISQV-high	270.00	410.00	218.00	Chapman et al. (1999)
Effects range low (ERL)	34.00	150.00	46.70	Long et al. (1995)
Effects range median (ERM)	270.00	410.00	218.00	Long et al. (1995)
Treshold effect level (TEL)	18.70	124.00	30.20	MacDonald et al. (1996)
Probable effect level (PEL)	108.20	271.00	112.20	MacDonald et al. (1996)
TEL	19.00	124.00	30.00	MacDonald et al. (2000)
PEL	108.00	271.00	112.00	MacDonald et al. (2000)
SQG (TEC)	32.00	121.00	35.90	Mao et al. (2019)
SGQ (PEC)	149.00	459.00	128.0	Mao et al. (2019)
Interim sediment quality guideline	18.70	124.00	30.20	ISQG (1995)
Probable effects level (PEL)	108.00	271.00	112.00	ISQG (1995)
Reference values	Cu	Zn	Pb	References
Pre-industrial reference level	50.00	175.00	70.00	Hakanson (1980)
Upper continental crust	28.00	67.00	17.00	Rudnick and Gao (2003)
Background continental crust	55.00	70.00	12.50	Taylor (1964)
Upper continental crust	25.00	71.00	17.00	Taylor and McLennan (1995)
Average sediment/Earth's crust	40.00	65.00	17.00	Taylor and McLennan (2008)
Background shale	45.00	95.00	20.00	Turekian and Wedepohl (1961)
Background upper continental crust	25.00	65.00	15.00	Wedepohl (2004)
Upper continental crust	14.30	52.00	17.00	Wedepohl (1995)
ASEAN-5 (N= 176)	0.09-3080 (84.2)	0.37-4950 (175)	0.07-2666 (76.0)	This study

Table S2: Values of concentration factors (Cf), ecological risk (ER), and potential ecological risk index (PERI) calculated from the present study based on the cited concentrations of Cu, Pb and Zn reported from ASEAN-5 countries.

	No.	Cf Cu	Cf Zn	Cf Pb	Cu ER	Zn ER	Pb ER	PERI	Cu %	Zn %	Pb %
Indonesia	1	0.15	0.01	0.06	0.73	0.01	0.32	1.07	68.7	1.1	30.3
	2	2.07	0.18	0.64	10.4	0.18	3.21	13.7	75.4	1.3	23.3
	3	5.80	1.53	25.8	29.0	1.53	129	159	18.2	1.0	80.8
	4	1.68	0.77	0.53	8.39	0.77	2.65	11.8	71.1	6.5	22.4
	5	4.06	2.35	1.35	20.3	2.35	6.76	29.4	69.0	8.0	23.0
	6	0.86	1.03	0.31	4.30	1.03	1.53	6.86	62.7	15.0	22.3
	7	31.3	24.2	157	157	24.2	784	965	16.2	2.5	81.3
	8	0.91	1.31	1.00	4.55	1.31	5.00	10.9	41.9	12.0	46.1
	9	1.75	1.83	1.65	8.74	1.83	8.24	18.8	46.5	9.7	43.8
	10	3.24	1.86	5.19	16.2	1.86	25.9	44.0	36.8	4.2	59.0
	11	1.15	0.12	0.72	5.77	0.12	3.59	9.47	60.9	1.2	37.9
	12	0.11	0.61	0.86	0.56	0.61	4.30	5.47	10.3	11.1	78.6
	13	0.97	1.68	4.99	4.84	1.68	25.0	31.5	15.4	5.3	79.3
	14	0.76	1.63	0.76	3.78	1.63	3.82	9.23	40.9	17.7	41.4
	15	7.48	16.25	6.24	37.4	16.3	31.2	84.8	44.1	19.2	36.7
	16	0.06	1.04	0.21	0.29	1.04	1.07	2.39	12.0	43.3	44.7
	17	5.22	9.57	3.12	26.1	9.57	15.6	51.3	50.9	18.7	30.4
	18	0.70	0.63	0.53	3.50	0.63	2.65	6.78	51.6	9.4	39.1
	19	1.93	3.59	1.62	9.67	3.59	8.11	21.4	45.2	16.8	38.0
	20	1.03	1.23	0.18	5.17	1.23	0.88	7.29	71.0	16.9	12.1
	21	24.8	21.7	12.4	124	21.7	61.8	208	59.8	10.5	29.7
	22	0.49	5.21	0.02	2.44	5.21	0.10	7.75	31.5	67.3	1.3
	23	0.77	13.1	0.23	3.85	13.1	1.14	18.1	21.3	72.4	6.3
	24	0.12	0.22	17.2	0.59	0.22	85.9	86.7	0.7	0.3	99.1
Thailand	1	0.21	0.67	1.18	1.05	0.67	5.88	7.60	13.8	8.9	77.4
	2	2.59	1.83	16.7	12.9	1.83	83.2	98.0	13.2	1.9	84.9
	3	4.90	4.81	24.9	24.5	4.81	125	154	15.9	3.1	81.0
	4	1.40	1.15	1.29	6.99	1.15	6.47	14.6	47.8	7.9	44.3
	5	6.99	1.35	0.94	35.0	1.35	4.71	41.0	85.2	3.3	11.5
	6	1.57	1.44	4.67	7.87	1.44	23.4	32.7	24.1	4.4	71.5
	7	1.88	1.73	5.71	9.41	1.73	28.5	39.7	23.7	4.4	71.9
	8	0.42	0.35	0.76	2.10	0.35	3.82	6.27	33.5	5.5	61.0
	9	2.52	2.44	2.12	12.6	2.44	10.6	25.6	49.1	9.5	41.3
	10	0.52	0.35	0.11	2.59	0.35	0.54	3.47	74.6	10.0	15.4
	11	0.23	0.18	0.56	1.14	0.18	2.81	4.14	27.6	4.3	68.0
	12	0.15	0.03	0.24	0.77	0.03	1.21	2.02	38.2	1.6	60.3
	13	1.77	1.52	2.28	8.85	1.52	11.4	21.8	40.7	7.0	52.4
	14	0.20	0.14	0.48	1.01	0.14	2.40	3.56	28.5	4.1	67.4
	15	0.96	0.89	1.53	4.81	0.89	7.64	13.3	36.0	6.7	57.3
	16	0.23	0.58	9.76	1.15	0.58	48.8	50.6	2.3	1.2	96.6
	17	1.31	0.77	18.5	6.54	0.77	92.4	99.7	6.6	0.8	92.7
	18	15.0	4.62	3.68	74.8	4.62	18.4	97.9	76.5	4.7	18.8
Vietnam	1	2.59	1.79	2.53	12.9	1.79	12.7	27.4	47.3	6.5	46.2
	2	21.6	95.2	21.2	108	95.2	106	309	34.9	30.8	34.3
	3	13.5	7.33	20.0	67.5	7.33	100	175	38.6	4.2	57.2
	4	1.40	0.77	0.94	6.99	0.77	4.71	12.5	56.1	6.2	37.7
	5	0.46	0.60	1.02	2.30	0.60	5.12	8.01	28.7	7.4	63.9
	6	2.00	1.77	1.99	10.0	1.77	9.94	21.7	46.1	8.1	45.8
	7	0.91	0.23	0.24	4.55	0.23	1.18	5.95	76.4	3.9	19.8

	8	2.10	1.81	2.41	10.5	1.81	12.1	24.4	43.1	7.4	49.5
	9	5.73	3.42	5.41	28.7	3.42	27.1	59.2	48.5	5.8	45.7
	10	0.98	1.25	1.06	4.90	1.25	5.29	11.4	42.8	10.9	46.3
	11	0.84	1.52	2.00	4.20	1.52	10.0	15.7	26.7	9.7	63.6
	12	1.27	1.23	1.94	6.33	1.23	9.71	17.3	36.7	7.1	56.2
	13	5.16	2.27	5.07	25.8	2.27	25.4	53.4	48.3	4.3	47.5
	14	1.40	0.77	1.59	6.99	0.77	7.94	15.7	44.5	4.9	50.6
	15	23.2	5.52	11.1	116	5.52	55.3	177	65.6	3.1	31.3
	16	1.15	1.40	1.00	5.77	1.40	5.00	12.2	47.4	11.5	41.1
	17	3.39	2.44	1.47	17.0	2.44	7.35	26.8	63.4	9.1	27.5
	18	1.82	2.17	1.53	9.09	2.17	7.65	18.9	48.1	11.5	40.4
	19	0.05	0.08	0.34	0.24	0.08	1.70	2.02	12.0	3.8	84.3
	20	6.63	9.46	7.06	33.1	9.46	35.3	77.9	42.5	12.1	45.3
	21	0.27	0.12	0.60	1.33	0.12	2.99	4.44	29.9	2.7	67.3
	22	2.92	2.29	4.11	14.6	2.29	20.6	37.43	39.0	6.1	54.9
	23	0.21	1.59	3.84	1.07	1.59	19.2	21.87	4.9	7.3	87.8
	24	0.64	4.08	5.01	3.20	4.08	25.1	32.34	9.9	12.6	77.5
East PM	1	2.04	1.39	3.23	10.2	1.39	16.2	27.75	36.8	5.0	58.2
	2	2.17	0.40	0.62	10.9	0.40	3.09	14.36	75.7	2.8	21.5
	3	2.03	0.43	0.69	10.1	0.43	3.44	14.0	72.4	3.1	24.6
	4	0.47	0.36	1.22	2.36	0.36	6.12	8.84	26.7	4.1	69.3
	5	2.62	0.18	2.61	13.1	0.18	13.0	26.3	49.8	0.7	49.6
	6	0.05	0.01	0.02	0.24	0.01	0.09	0.34	70.8	4.2	25.0
	7	1.35	1.18	1.21	6.75	1.18	6.03	14.0	48.4	8.4	43.2
	8	1.70	3.07	1.72	8.50	3.07	8.62	20.2	42.1	15.2	42.7
	9	0.03	0.04	0.09	0.13	0.04	0.47	0.64	20.6	6.4	73.0
	10	1.12	0.92	2.48	5.59	0.92	12.4	18.9	29.6	4.8	65.6
	11	1.76	1.22	3.85	8.81	1.22	19.3	29.3	30.1	4.2	65.8
	12	2.56	0.79	1.16	12.8	0.79	5.82	19.4	65.9	4.1	30.0
	13	0.80	0.63	1.33	3.99	0.63	6.65	11.3	35.4	5.6	59.0
	14	0.98	0.79	3.66	4.92	0.79	18.3	24.0	20.5	3.3	76.2
East Malaysia	1	1.33	0.79	2.41	6.64	0.79	12.1	19.5	34.1	4.0	61.9
	2	1.96	1.10	3.06	9.79	1.10	15.3	26.2	37.4	4.2	58.4
	3	2.01	0.34	0.62	10.1	0.34	3.09	13.5	74.6	2.5	22.9
	4	4.99	0.54	1.16	24.9	0.54	5.79	31.3	79.8	1.7	18.5
	5	17.3	8.67	3.15	86.7	8.67	15.8	111	78.0	7.8	14.2
	6	40.8	1.18	1.38	204	1.18	6.91	212	96.2	0.6	3.3
	7	0.45	0.42	0.15	2.27	0.42	0.77	3.47	65.5	12.2	22.3
	8	0.56	0.31	0.26	2.82	0.31	1.31	4.44	63.6	6.9	29.5
	9	27.3	1.33	1.92	137	1.33	9.59	148	92.6	0.9	6.5
	10	1.39	1.06	0.44	6.96	1.06	2.22	10.2	68.0	10.3	21.7
	11	0.01	0.12	0.00	0.03	0.12	0.02	0.17	18.2	69.8	11.9
Straits of Johore	1	0.31	0.50	1.81	1.54	0.50	9.06	11.1	13.9	4.5	81.6
	2	0.76	1.32	1.55	3.78	1.32	7.76	12.9	29.4	10.2	60.4
	3	6.50	4.44	4.11	32.5	4.44	20.6	57.5	56.5	7.7	35.8
	4	4.04	4.05	3.09	20.2	4.05	15.5	39.7	50.9	10.2	38.9
	5	7.69	3.46	1.98	38.5	3.46	9.88	51.8	74.2	6.7	19.1
	6	2.00	2.63	1.98	10.0	2.63	9.91	22.6	44.4	11.7	44.0
West PM	1	0.07	0.15	0.35	0.35	0.15	1.76	2.27	15.4	6.8	77.8
	2	1.82	1.46	2.06	9.09	1.46	10.3	20.9	43.6	7.0	49.4
	3	2.80	1.92	2.82	14.0	1.92	14.1	30.0	46.6	6.4	47.0
	4	0.65	1.41	1.22	3.25	1.41	6.12	10.8	30.2	13.1	56.7
	5	0.97	2.11	1.94	4.83	2.11	9.71	16.6	29.0	12.7	58.3
	6	0.70	26.9	2.65	3.49	26.9	13.2	43.7	8.0	61.7	30.3

	7	0.49	0.75	0.65	2.45	0.75	3.24	6.43	38.0	11.7	50.3
	8	0.91	1.75	2.12	4.55	1.75	10.59	16.88	26.9	10.4	62.7
	9	0.14	0.04	0.01	0.70	0.04	0.03	0.77	91.2	5.0	3.8
	10	10.07	9.29	6.88	50.4	9.29	34.4	94.1	53.5	9.9	36.6
	11	1.61	1.06	0.94	8.04	1.06	4.71	13.8	58.3	7.7	34.1
	12	0.70	0.58	1.31	3.49	0.58	6.53	10.60	32.9	5.5	61.6
	13	4.44	9.87	2.67	22.2	9.87	13.3	45.4	48.9	21.7	29.4
	14	0.02	0.08	0.21	0.09	0.08	1.06	1.22	7.2	6.3	86.5
	15	0.97	1.52	1.49	4.83	1.52	7.46	13.8	35.0	11.0	54.0
	16	0.03	0.06	0.06	0.14	0.06	0.28	0.48	29.0	12.4	58.6
	17	22.0	5.88	4.11	110	5.88	20.5	137	80.7	4.3	15.0
	18	0.24	0.83	0.97	1.20	0.83	4.85	6.88	17.4	12.0	70.5
	19	10.8	6.23	3.99	53.9	6.23	19.9	80.1	67.3	7.8	24.9
	20	3.01	3.25	3.30	15.0	3.25	16.5	34.8	43.2	9.3	47.4
	21	38.5	5.69	23.8	193	5.69	119	317	60.7	1.8	37.5
	22	61.3	6.35	3.38	307	6.35	16.9	330	93.0	1.9	5.1
	23	71.3	9.31	74.5	356	9.31	373	738	48.3	1.3	50.5
	24	0.46	0.97	1.53	2.32	0.97	7.65	10.9	21.2	8.8	69.9
	25	8.60	6.46	13.4	43.0	6.46	67.0	116	36.9	5.5	57.5
	26	2.30	6.10	1.78	11.5	6.10	8.88	26.5	43.4	23.0	33.5
	27	0.42	0.67	3.75	2.10	0.67	18.73	21.5	9.8	3.1	87.1
	28	2.24	10.2	3.58	11.2	10.2	17.9	39.2	28.5	26.0	45.5
	29	0.37	0.89	1.79	1.85	0.89	8.94	11.7	15.8	7.6	76.5
	30	3.77	3.98	3.66	18.9	3.98	18.3	41.2	45.8	9.7	44.5
	31	0.36	0.90	0.18	1.82	0.90	0.90	3.62	50.2	24.9	24.9
	32	0.86	1.49	0.48	4.30	1.49	2.40	8.20	52.5	18.2	29.3
	33	0.52	0.44	1.31	2.59	0.44	6.56	9.59	27.0	4.6	68.4
	34	1.93	1.89	4.71	9.65	1.89	23.5	35.1	27.5	5.4	67.1
	35	0.33	0.65	0.94	1.67	0.65	4.68	7.00	23.9	9.2	66.8
	36	2.30	6.10	3.62	11.5	6.10	18.1	35.7	32.2	17.1	50.7
	37	0.71	1.71	0.60	3.57	1.71	3.00	8.27	43.1	20.6	36.3
	38	8.39	9.31	7.41	42.0	9.31	37.1	88.3	47.5	10.5	42.0
	39	2.43	1.81	2.19	12.1	1.81	10.9	24.9	48.7	7.3	44.0
	40	1.34	0.99	1.59	6.68	0.99	7.94	15.6	42.8	6.3	50.9
	41	4.20	3.10	3.58	20.9	3.10	17.9	42.0	50.0	7.4	42.6
	42	14.1	12.5	13.2	70.6	12.5	66.2	149	47.3	8.4	44.3
	43	3.01	1.61	2.09	15.0	1.61	10.4	27.1	55.5	5.9	38.5
	44	0.40	0.69	1.79	2.00	0.69	8.95	11.6	17.2	5.9	76.9
	45	1.22	0.98	3.50	6.09	0.98	17.5	24.6	24.8	4.0	71.2
	46	0.51	1.18	1.38	2.56	1.18	6.92	10.7	24.0	11.1	65.0
	47	2.51	2.33	4.51	12.6	2.33	22.6	37.4	33.5	6.2	60.2
	48	0.19	0.57	0.91	0.94	0.57	4.56	6.07	15.4	9.4	75.2
	49	0.01	0.01	0.03	0.06	0.01	0.16	0.23	26.8	3.0	70.2
	50	0.48	0.93	0.92	2.38	0.93	4.59	7.89	30.1	11.7	58.1
	51	3.28	19.7	4.64	16.4	19.7	23.2	59.3	27.7	33.2	39.1
	52	3.70	5.23	3.77	18.5	5.23	18.9	42.6	43.4	12.3	44.3
	53	0.39	0.56	1.49	1.95	0.56	7.46	9.98	19.6	5.7	74.8
	54	2.01	2.51	10.2	10.0	2.51	50.8	63.3	15.9	4.0	80.2
	55	0.22	1.41	0.47	1.12	1.41	2.35	4.88	22.9	28.8	48.2
	56	0.71	2.41	1.51	3.57	2.41	7.56	13.5	26.3	17.8	55.8
	57	0.13	0.17	0.46	0.67	0.17	2.28	3.12	21.5	5.5	73.0
	58	27.0	1.98	3.72	135	1.98	18.6	156	86.8	1.3	11.9
	59	0.73	1.07	2.83	3.67	1.07	14.2	18.9	19.5	5.7	74.9
	60	1.22	1.63	3.78	6.08	1.63	18.9	26.6	22.8	6.1	71.0
	61	0.25	1.47	1.69	1.24	1.47	8.47	11.2	11.1	13.2	75.7

	62	0.24	0.58	0.52	1.22	0.58	2.61	4.42	27.7	13.1	59.2
	63	1.16	1.53	1.72	5.80	1.53	8.61	15.9	36.4	9.6	54.0
	64	0.07	4.14	0.52	0.35	4.14	2.60	7.09	4.9	58.4	36.6
	65	0.74	4.98	1.68	3.71	4.98	8.41	17.1	21.7	29.1	49.2
	66	0.66	0.43	2.20	3.28	0.43	11.0	14.7	22.3	2.9	74.8
	67	0.67	1.39	0.89	3.34	1.39	4.44	9.17	36.4	15.1	48.4
	68	4.54	5.85	3.50	22.7	5.85	17.5	46.0	49.3	12.7	38.0
Philippines	1	6.99	2.19	0.61	35.0	2.19	3.06	40.2	86.9	5.5	7.6
	2	2.24	1.15	0.35	11.2	1.15	1.76	14.1	79.3	8.2	12.5
	3	8.25	6.33	5.59	41.3	6.33	27.9	75.5	54.6	8.4	37.0
	4	49.4	2.52	2.53	247	2.52	12.7	262	94.2	1.0	4.8
	5	215	5.31	3.29	1077	5.31	16.5	1099	98.0	0.5	1.5
	6	1.60	0.96	0.43	8.01	0.96	2.15	11.1	72.0	8.7	19.3
	7	2.70	1.85	1.12	13.5	1.85	5.59	20.9	64.5	8.8	26.7
	8	3.97	1.43	0.51	19.8	1.43	2.56	23.8	83.2	6.0	10.7
	9	6.31	2.35	1.56	31.6	2.35	7.82	41.7	75.6	5.6	18.7
	10	4.67	2.15	1.20	23.4	2.15	6.00	31.5	74.1	6.8	19.0
	11	10.3	4.83	3.82	51.4	4.83	19.1	75.4	68.3	6.4	25.3
**Sediment Quality Guidelines**		Cf Cu	Cf Zn	Cf Pb	Er Cu	Er Zn	Er Pb	PERI	Cu %	Zn %	Pb %
ISQV-low	ISQV-low	4.55	3.85	4.41	22.73	3.85	22.06	48.63	46.7	7.9	45.4
ISQV-high	ISQV-high	18.88	7.88	12.82	94.41	7.88	64.12	166.41	56.7	4.7	38.5
Effects range low (ERL)	ERL	2.38	2.88	2.75	11.89	2.88	13.74	28.51	41.7	10.1	48.2
Effects range median (ERM)	ERM	18.88	7.88	12.82	94.41	7.88	64.12	166.41	56.7	4.7	38.5
Treshold effect level (TEL)	TEL	1.31	2.38	1.78	6.54	2.38	8.88	17.81	36.7	13.4	49.9
Probable effect level (PEL)	PEL	7.57	5.21	6.60	37.83	5.21	33.00	76.04	49.8	6.9	43.4
TEL	TEL	1.33	2.38	1.76	6.64	2.38	8.82	17.85	37.2	13.4	49.4
PEL	PEL	7.55	5.21	6.59	37.76	5.21	32.94	75.91	49.7	6.9	43.4
SQG (TEC)	SQG (TEC)	2.24	2.33	2.11	11.19	2.33	10.56	24.07	46.5	9.7	43.9
SGQ (PEC)	SGQ (PEC)	10.4	8.83	7.53	52.10	8.83	37.65	98.57	52.9	9.0	38.2
Interim sediment quality guideline	ISQG	1.31	2.38	1.78	6.54	2.38	8.88	17.81	36.7	13.4	49.9
Probable effects level (PEL)	PEL	7.55	5.21	6.59	37.76	5.21	32.94	75.91	49.7	6.9	43.4
**Reference values**											
Pre-industrial reference level	1980	3.50	3.37	4.12	17.48	3.37	20.59	41.44	42.2	8.1	49.7
Upper continental crust	2003	1.96	1.29	1.00	9.79	1.29	5.00	16.08	60.9	8.0	31.1
Background continental crust	1964	3.85	1.35	0.74	19.23	1.35	3.68	24.25	79.3	5.6	15.2
Upper continental crust	1994	1.75	1.37	1.00	8.74	1.37	5.00	15.11	57.9	9.0	33.1
Average sediment/Earth's crust	2008	2.80	1.25	1.00	13.99	1.25	5.00	20.24	69.1	6.2	24.7

Background shale	1961	3.15	1.83	1.18	15.73	1.83	5.88	23.44	67.1	7.8	25.1
Upper continental crust	2004	1.75	1.25	0.88	8.74	1.25	4.41	14.40	60.7	8.7	30.6
Upper continental crust	1995	1.00	1.00	1.00	5.00	1.00	5.00	11.00	45.5	9.1	45.5

Table S3: Values of hazard quotient ingestion (HQ_{ing}), hazard quotient dermal (HQ_{dermal}) and hazard index (HI) of Cu for children and adults from the present study.

	No.	Children			Adults		
Cu		HQ_{ing}	HQ_{dermal}	HI	HQ_{ing}	HQ_{dermal}	HI
Indonesia	1	6.88E-04	3.67E-06	6.92E-04	9.23E-05	9.37E-06	1.02E-04
	2	9.70E-03	5.17E-05	9.75E-03	1.30E-03	1.32E-04	1.43E-03
	3	2.72E-02	1.45E-04	2.73E-02	3.65E-03	3.70E-04	4.01E-03
	4	7.87E-03	4.19E-05	7.91E-03	1.06E-03	1.07E-04	1.16E-03
	5	1.90E-02	1.01E-04	1.91E-02	2.55E-03	2.59E-04	2.81E-03
	6	4.03E-03	2.15E-05	4.05E-03	5.41E-04	5.49E-05	5.96E-04
	7	1.47E-01	7.83E-04	1.48E-01	1.97E-02	2.00E-03	2.17E-02
	8	4.26E-03	2.27E-05	4.28E-03	5.72E-04	5.80E-05	6.30E-04
	9	8.19E-03	4.37E-05	8.24E-03	1.10E-03	1.12E-04	1.21E-03
	10	1.52E-02	8.09E-05	1.53E-02	2.04E-03	2.07E-04	2.24E-03
	11	5.41E-03	2.88E-05	5.44E-03	7.25E-04	7.36E-05	7.99E-04
	12	5.28E-04	2.81E-06	5.30E-04	7.08E-05	7.19E-06	7.80E-05
	13	4.54E-03	2.42E-05	4.56E-03	6.09E-04	6.18E-05	6.70E-04
	14	3.54E-03	1.89E-05	3.56E-03	4.75E-04	4.82E-05	5.23E-04
	15	3.51E-02	1.87E-04	3.53E-02	4.70E-03	4.78E-04	5.18E-03
	16	2.69E-04	1.43E-06	2.70E-04	3.61E-05	3.66E-06	3.97E-05
	17	2.45E-02	1.31E-04	2.46E-02	3.28E-03	3.33E-04	3.62E-03
	18	3.28E-03	1.75E-05	3.29E-03	4.40E-04	4.46E-05	4.84E-04
	19	9.06E-03	4.83E-05	9.11E-03	1.22E-03	1.23E-04	1.34E-03
	20	4.85E-03	2.59E-05	4.88E-03	6.51E-04	6.61E-05	7.17E-04
	21	1.16E-01	6.20E-04	1.17E-01	1.56E-02	1.58E-03	1.72E-02
	22	2.29E-03	1.22E-05	2.30E-03	3.07E-04	3.12E-05	3.38E-04
	23	3.60E-03	1.92E-05	3.62E-03	4.84E-04	4.91E-05	5.33E-04
	24	5.57E-04	2.97E-06	5.60E-04	7.47E-05	7.59E-06	8.23E-05
Thailand	1	9.83E-04	5.24E-06	9.88E-04	1.32E-04	1.34E-05	1.45E-04
	2	1.21E-02	6.47E-05	1.22E-02	1.63E-03	1.65E-04	1.79E-03
	3	2.29E-02	1.22E-04	2.31E-02	3.08E-03	3.12E-04	3.39E-03
	4	6.55E-03	3.50E-05	6.59E-03	8.79E-04	8.93E-05	9.69E-04
	5	3.28E-02	1.75E-04	3.29E-02	4.40E-03	4.46E-04	4.84E-03
	6	7.37E-03	3.93E-05	7.41E-03	9.89E-04	1.00E-04	1.09E-03
	7	8.82E-03	4.70E-05	8.86E-03	1.18E-03	1.20E-04	1.30E-03
	8	1.97E-03	1.05E-05	1.98E-03	2.64E-04	2.68E-05	2.91E-04
	9	1.18E-02	6.29E-05	1.19E-02	1.58E-03	1.61E-04	1.74E-03
	10	2.43E-03	1.30E-05	2.44E-03	3.26E-04	3.31E-05	3.59E-04
	11	1.07E-03	5.72E-06	1.08E-03	1.44E-04	1.46E-05	1.58E-04
	12	7.21E-04	3.85E-06	7.25E-04	9.67E-05	9.82E-06	1.07E-04
	13	8.29E-03	4.42E-05	8.34E-03	1.11E-03	1.13E-04	1.23E-03
	14	9.50E-04	5.07E-06	9.55E-04	1.28E-04	1.29E-05	1.40E-04
	15	4.51E-03	2.40E-05	4.53E-03	6.05E-04	6.14E-05	6.66E-04
	16	1.08E-03	5.77E-06	1.09E-03	1.45E-04	1.47E-05	1.60E-04
	17	6.13E-03	3.27E-05	6.16E-03	8.22E-04	8.35E-05	9.06E-04
	18	7.01E-02	3.74E-04	7.05E-02	9.41E-03	9.55E-04	1.04E-02
Vietnam	1	1.21E-02	6.47E-05	1.22E-02	1.63E-03	1.65E-04	1.79E-03
	2	1.01E-01	5.40E-04	1.02E-01	1.36E-02	1.38E-03	1.50E-02
	3	6.32E-02	3.37E-04	6.36E-02	8.49E-03	8.61E-04	9.35E-03

	4	6.55E-03	3.50E-05	6.59E-03	8.79E-04	8.93E-05	9.69E-04
	5	2.16E-03	1.15E-05	2.17E-03	2.89E-04	2.94E-05	3.19E-04
	6	9.37E-03	5.00E-05	9.42E-03	1.26E-03	1.28E-04	1.39E-03
	7	4.26E-03	2.27E-05	4.28E-03	5.72E-04	5.80E-05	6.30E-04
	8	9.83E-03	5.24E-05	9.88E-03	1.32E-03	1.34E-04	1.45E-03
	9	2.69E-02	1.43E-04	2.70E-02	3.61E-03	3.66E-04	3.97E-03
	10	4.59E-03	2.45E-05	4.61E-03	6.16E-04	6.25E-05	6.78E-04
	11	3.93E-03	2.10E-05	3.95E-03	5.28E-04	5.36E-05	5.81E-04
	12	5.93E-03	3.16E-05	5.96E-03	7.96E-04	8.08E-05	8.77E-04
	13	2.42E-02	1.29E-04	2.43E-02	3.24E-03	3.29E-04	3.57E-03
	14	6.55E-03	3.50E-05	6.59E-03	8.79E-04	8.93E-05	9.69E-04
	15	1.09E-01	5.80E-04	1.09E-01	1.46E-02	1.48E-03	1.61E-02
	16	5.41E-03	2.88E-05	5.44E-03	7.25E-04	7.36E-05	7.99E-04
	17	1.59E-02	8.48E-05	1.60E-02	2.13E-03	2.16E-04	2.35E-03
	18	8.52E-03	4.54E-05	8.57E-03	1.14E-03	1.16E-04	1.26E-03
	19	2.26E-04	1.21E-06	2.27E-04	3.03E-05	3.08E-06	3.34E-05
	20	3.11E-02	1.66E-04	3.12E-02	4.17E-03	4.23E-04	4.59E-03
	21	1.25E-03	6.64E-06	1.25E-03	1.67E-04	1.70E-05	1.84E-04
	22	1.37E-02	7.29E-05	1.37E-02	1.83E-03	1.86E-04	2.02E-03
	23	1.01E-03	5.37E-06	1.01E-03	1.35E-04	1.37E-05	1.49E-04
	24	3.00E-03	1.60E-05	3.01E-03	4.02E-04	4.08E-05	4.43E-04
East PM	1	9.57E-03	5.10E-05	9.62E-03	1.28E-03	1.30E-04	1.41E-03
	2	1.02E-02	5.44E-05	1.02E-02	1.37E-03	1.39E-04	1.51E-03
	3	9.50E-03	5.07E-05	9.55E-03	1.28E-03	1.29E-04	1.40E-03
	4	2.21E-03	1.18E-05	2.22E-03	2.96E-04	3.01E-05	3.26E-04
	5	1.23E-02	6.54E-05	1.23E-02	1.64E-03	1.67E-04	1.81E-03
	6	2.26E-04	1.21E-06	2.27E-04	3.03E-05	3.08E-06	3.34E-05
	7	6.32E-03	3.37E-05	6.36E-03	8.49E-04	8.61E-05	9.35E-04
	8	7.96E-03	4.25E-05	8.01E-03	1.07E-03	1.08E-04	1.18E-03
	9	1.25E-04	6.64E-07	1.25E-04	1.67E-05	1.70E-06	1.84E-05
	10	5.24E-03	2.80E-05	5.27E-03	7.04E-04	7.14E-05	7.75E-04
	11	8.26E-03	4.40E-05	8.30E-03	1.11E-03	1.12E-04	1.22E-03
	12	1.20E-02	6.40E-05	1.21E-02	1.61E-03	1.63E-04	1.77E-03
	13	3.74E-03	1.99E-05	3.76E-03	5.01E-04	5.09E-05	5.52E-04
	14	4.61E-03	2.46E-05	4.64E-03	6.19E-04	6.28E-05	6.81E-04
East Malaysia	1	6.23E-03	3.32E-05	6.26E-03	8.35E-04	8.48E-05	9.20E-04
	2	9.18E-03	4.89E-05	9.22E-03	1.23E-03	1.25E-04	1.36E-03
	3	9.44E-03	5.03E-05	9.49E-03	1.27E-03	1.29E-04	1.39E-03
	4	2.34E-02	1.25E-04	2.35E-02	3.13E-03	3.18E-04	3.45E-03
	5	8.13E-02	4.33E-04	8.17E-02	1.09E-02	1.11E-03	1.20E-02
	6	1.91E-01	1.02E-03	1.92E-01	2.57E-02	2.60E-03	2.83E-02
	7	2.13E-03	1.13E-05	2.14E-03	2.85E-04	2.90E-05	3.14E-04
	8	2.64E-03	1.41E-05	2.66E-03	3.55E-04	3.60E-05	3.91E-04
	9	1.28E-01	6.83E-04	1.29E-01	1.72E-02	1.74E-03	1.89E-02
	10	6.52E-03	3.48E-05	6.56E-03	8.75E-04	8.88E-05	9.64E-04
	11	2.95E-05	1.57E-07	2.97E-05	3.96E-06	4.02E-07	4.36E-06
Straits of Johore	1	1.44E-03	7.69E-06	1.45E-03	1.93E-04	1.96E-05	2.13E-04

	2	3.54E-03	1.89E-05	3.56E-03	4.75E-04	4.82E-05	5.23E-04
	3	3.04E-02	1.62E-04	3.06E-02	4.08E-03	4.15E-04	4.50E-03
	4	1.90E-02	1.01E-04	1.91E-02	2.54E-03	2.58E-04	2.80E-03
	5	3.60E-02	1.92E-04	3.62E-02	4.84E-03	4.91E-04	5.33E-03
	6	9.37E-03	5.00E-05	9.42E-03	1.26E-03	1.28E-04	1.39E-03
West PM	1	3.28E-04	1.75E-06	3.29E-04	4.40E-05	4.46E-06	4.84E-05
	2	8.52E-03	4.54E-05	8.57E-03	1.14E-03	1.16E-04	1.26E-03
	3	1.31E-02	6.99E-05	1.32E-02	1.76E-03	1.79E-04	1.94E-03
	4	3.05E-03	1.63E-05	3.06E-03	4.09E-04	4.15E-05	4.50E-04
	5	4.52E-03	2.41E-05	4.55E-03	6.07E-04	6.16E-05	6.68E-04
	6	3.27E-03	1.75E-05	3.29E-03	4.39E-04	4.46E-05	4.84E-04
	7	2.29E-03	1.22E-05	2.31E-03	3.08E-04	3.12E-05	3.39E-04
	8	4.26E-03	2.27E-05	4.28E-03	5.72E-04	5.80E-05	6.30E-04
	9	6.55E-04	3.50E-06	6.59E-04	8.79E-05	8.93E-06	9.69E-05
	10	4.72E-02	2.52E-04	4.74E-02	6.33E-03	6.43E-04	6.97E-03
	11	7.54E-03	4.02E-05	7.58E-03	1.01E-03	1.03E-04	1.11E-03
	12	3.27E-03	1.75E-05	3.29E-03	4.39E-04	4.46E-05	4.84E-04
	13	2.08E-02	1.11E-04	2.09E-02	2.79E-03	2.83E-04	3.07E-03
	14	8.19E-05	4.37E-07	8.24E-05	1.10E-05	1.12E-06	1.21E-05
	15	4.53E-03	2.42E-05	4.55E-03	6.08E-04	6.17E-05	6.69E-04
	16	1.31E-04	6.99E-07	1.32E-04	1.76E-05	1.79E-06	1.94E-05
	17	1.03E-01	5.51E-04	1.04E-01	1.39E-02	1.41E-03	1.53E-02
	18	1.12E-03	6.00E-06	1.13E-03	1.51E-04	1.53E-05	1.66E-04
	19	5.05E-02	2.70E-04	5.08E-02	6.78E-03	6.88E-04	7.47E-03
	20	1.41E-02	7.52E-05	1.42E-02	1.89E-03	1.92E-04	2.08E-03
	21	1.81E-01	9.63E-04	1.82E-01	2.42E-02	2.46E-03	2.67E-02
	22	2.87E-01	1.53E-03	2.89E-01	3.86E-02	3.91E-03	4.25E-02
	23	3.34E-01	1.78E-03	3.36E-01	4.48E-02	4.55E-03	4.94E-02
	24	2.18E-03	1.16E-05	2.19E-03	2.92E-04	2.96E-05	3.22E-04
	25	4.03E-02	2.15E-04	4.05E-02	5.41E-03	5.49E-04	5.96E-03
	26	1.08E-02	5.75E-05	1.08E-02	1.45E-03	1.47E-04	1.59E-03
	27	1.97E-03	1.05E-05	1.98E-03	2.64E-04	2.68E-05	2.91E-04
	28	1.05E-02	5.59E-05	1.05E-02	1.41E-03	1.43E-04	1.55E-03
	29	1.73E-03	9.25E-06	1.74E-03	2.33E-04	2.36E-05	2.56E-04
	30	1.77E-02	9.42E-05	1.78E-02	2.37E-03	2.41E-04	2.61E-03
	31	1.70E-03	9.09E-06	1.71E-03	2.29E-04	2.32E-05	2.52E-04
	32	4.03E-03	2.15E-05	4.05E-03	5.41E-04	5.49E-05	5.96E-04
	33	2.43E-03	1.29E-05	2.44E-03	3.25E-04	3.30E-05	3.58E-04
	34	9.04E-03	4.82E-05	9.09E-03	1.21E-03	1.23E-04	1.34E-03
	35	1.57E-03	8.37E-06	1.58E-03	2.11E-04	2.14E-05	2.32E-04
	36	1.08E-02	5.75E-05	1.08E-02	1.45E-03	1.47E-04	1.59E-03
	37	3.34E-03	1.78E-05	3.36E-03	4.48E-04	4.55E-05	4.94E-04
	38	3.93E-02	2.10E-04	3.95E-02	5.28E-03	5.36E-04	5.81E-03
	39	1.14E-02	6.07E-05	1.14E-02	1.53E-03	1.55E-04	1.68E-03
	40	6.26E-03	3.34E-05	6.29E-03	8.40E-04	8.52E-05	9.25E-04
	41	1.97E-02	1.05E-04	1.98E-02	2.64E-03	2.68E-04	2.91E-03
	42	6.62E-02	3.53E-04	6.66E-02	8.88E-03	9.01E-04	9.78E-03
	43	1.41E-02	7.52E-05	1.42E-02	1.89E-03	1.92E-04	2.08E-03
	44	1.87E-03	1.00E-05	1.88E-03	2.52E-04	2.55E-05	2.77E-04

	45	5.71E-03	3.05E-05	5.74E-03	7.66E-04	7.78E-05	8.44E-04
	46	2.40E-03	1.28E-05	2.41E-03	3.21E-04	3.26E-05	3.54E-04
	47	1.18E-02	6.28E-05	1.18E-02	1.58E-03	1.60E-04	1.74E-03
	48	8.78E-04	4.68E-06	8.83E-04	1.18E-04	1.20E-05	1.30E-04
	49	5.90E-05	3.15E-07	5.93E-05	7.91E-06	8.03E-07	8.72E-06
	50	2.23E-03	1.19E-05	2.24E-03	2.99E-04	3.03E-05	3.29E-04
	51	1.54E-02	8.20E-05	1.54E-02	2.06E-03	2.09E-04	2.27E-03
	52	1.73E-02	9.25E-05	1.74E-02	2.33E-03	2.36E-04	2.56E-03
	53	1.83E-03	9.77E-06	1.84E-03	2.46E-04	2.49E-05	2.71E-04
	54	9.41E-03	5.02E-05	9.46E-03	1.26E-03	1.28E-04	1.39E-03
	55	1.05E-03	5.59E-06	1.05E-03	1.41E-04	1.43E-05	1.55E-04
	56	3.34E-03	1.78E-05	3.36E-03	4.48E-04	4.55E-05	4.94E-04
	57	6.30E-04	3.36E-06	6.33E-04	8.45E-05	8.58E-06	9.31E-05
	58	1.27E-01	6.76E-04	1.27E-01	1.70E-02	1.73E-03	1.87E-02
	59	3.44E-03	1.84E-05	3.46E-03	4.62E-04	4.69E-05	5.09E-04
	60	5.70E-03	3.04E-05	5.73E-03	7.65E-04	7.76E-05	8.42E-04
	61	1.16E-03	6.20E-06	1.17E-03	1.56E-04	1.58E-05	1.72E-04
	62	1.15E-03	6.12E-06	1.15E-03	1.54E-04	1.56E-05	1.70E-04
	63	5.43E-03	2.90E-05	5.46E-03	7.29E-04	7.40E-05	8.03E-04
	64	3.28E-04	1.75E-06	3.29E-04	4.40E-05	4.46E-06	4.84E-05
	65	3.47E-03	1.85E-05	3.49E-03	4.66E-04	4.73E-05	5.13E-04
	66	3.07E-03	1.64E-05	3.09E-03	4.12E-04	4.19E-05	4.54E-04
	67	3.13E-03	1.67E-05	3.15E-03	4.20E-04	4.27E-05	4.63E-04
	68	2.13E-02	1.13E-04	2.14E-02	2.85E-03	2.90E-04	3.14E-03
Philippines	1	3.28E-02	1.75E-04	3.29E-02	4.40E-03	4.46E-04	4.84E-03
	2	1.05E-02	5.59E-05	1.05E-02	1.41E-03	1.43E-04	1.55E-03
	3	3.87E-02	2.06E-04	3.89E-02	5.19E-03	5.27E-04	5.71E-03
	4	2.31E-01	1.23E-03	2.33E-01	3.10E-02	3.15E-03	3.42E-02
	5	1.01E+00	5.38E-03	1.01E+00	1.35E-01	1.37E-02	1.49E-01
	6	7.50E-03	4.00E-05	7.54E-03	1.01E-03	1.02E-04	1.11E-03
	7	1.26E-02	6.75E-05	1.27E-02	1.70E-03	1.72E-04	1.87E-03
	8	1.86E-02	9.91E-05	1.87E-02	2.49E-03	2.53E-04	2.75E-03
	9	2.96E-02	1.58E-04	2.98E-02	3.97E-03	4.03E-04	4.37E-03
	10	2.19E-02	1.17E-04	2.20E-02	2.94E-03	2.98E-04	3.24E-03
	11	4.82E-02	2.57E-04	4.85E-02	6.47E-03	6.56E-04	7.12E-03
	ISQV-low	2.13E-02	1.14E-04	2.14E-02	2.86E-03	2.90E-04	3.15E-03
	ISQV-high	8.85E-02	4.72E-04	8.90E-02	1.19E-02	1.20E-03	1.31E-02
	ERL	1.11E-02	5.94E-05	1.12E-02	1.49E-03	1.52E-04	1.65E-03
	ERM	8.85E-02	4.72E-04	8.90E-02	1.19E-02	1.20E-03	1.31E-02
	TEL	6.13E-03	3.27E-05	6.16E-03	8.22E-04	8.35E-05	9.06E-04
	PEL	3.55E-02	1.89E-04	3.56E-02	4.76E-03	4.83E-04	5.24E-03
	TEL	6.23E-03	3.32E-05	6.26E-03	8.35E-04	8.48E-05	9.20E-04
	PEL	3.54E-02	1.89E-04	3.56E-02	4.75E-03	4.82E-04	5.23E-03
	SQG (TEC)	1.05E-02	5.59E-05	1.05E-02	1.41E-03	1.43E-04	1.55E-03

	SGQ (PEC)	4.88E-02	2.60E-04	4.91E-02	6.55E-03	6.65E-04	7.22E-03
	ISQG	6.13E-03	3.27E-05	6.16E-03	8.22E-04	8.35E-05	9.06E-04
	PEL	3.54E-02	1.89E-04	3.56E-02	4.75E-03	4.82E-04	5.23E-03
	1980	1.64E-02	8.74E-05	1.65E-02	2.20E-03	2.23E-04	2.42E-03
	2003	9.18E-03	4.89E-05	9.22E-03	1.23E-03	1.25E-04	1.36E-03
	1964	1.80E-02	9.61E-05	1.81E-02	2.42E-03	2.45E-04	2.66E-03
	1994	8.19E-03	4.37E-05	8.24E-03	1.10E-03	1.12E-04	1.21E-03
	2008	1.31E-02	6.99E-05	1.32E-02	1.76E-03	1.79E-04	1.94E-03
	1961	1.47E-02	7.87E-05	1.48E-02	1.98E-03	2.01E-04	2.18E-03
	1995	4.69E-03	2.50E-05	4.71E-03	6.29E-04	6.38E-05	6.93E-04
	2004	8.19E-03	4.37E-05	8.24E-03	1.10E-03	1.12E-04	1.21E-03

Table S4: Values of hazard quotient ingestion (HQ_{ing}), hazard quotient dermal (HQ_{dermal}) and hazard index (HI) of Pb for children and adults from the present study.

	No.	Children			Adult		
Pb		HQ_{ing}	HQ_{dermal}	HI	HQ_{ing}	HQ_{dermal}	HI
Indonesia	1	4.07E-03	4.39E-05	4.12E-03	5.47E-04	1.12E-04	6.59E-04
	2	4.04E-02	4.35E-04	4.08E-02	5.42E-03	1.11E-03	6.53E-03
	3	**1.62E+00**	1.75E-02	**1.64E+00**	2.18E-01	4.47E-02	2.62E-01
	4	3.33E-02	3.60E-04	3.37E-02	4.47E-03	9.18E-04	5.39E-03
	5	8.52E-02	9.19E-04	8.61E-02	1.14E-02	2.35E-03	1.38E-02
	6	1.93E-02	2.08E-04	1.95E-02	2.58E-03	5.30E-04	3.11E-03
	7	**9.87E+00**	1.07E-01	**9.98E+00**	1.32E+00	2.72E-01	1.60E+00
	8	6.30E-02	6.79E-04	6.36E-02	8.45E-03	1.73E-03	1.02E-02
	9	1.04E-01	1.12E-03	1.05E-01	1.39E-02	2.86E-03	1.68E-02
	10	3.27E-01	3.52E-03	3.30E-01	4.38E-02	9.00E-03	5.28E-02
	11	4.52E-02	4.87E-04	4.57E-02	6.06E-03	1.24E-03	7.31E-03
	12	5.42E-02	5.84E-04	5.48E-02	7.27E-03	1.49E-03	8.76E-03
	13	3.14E-01	3.39E-03	3.18E-01	4.22E-02	8.66E-03	5.08E-02
	14	4.81E-02	5.19E-04	4.87E-02	6.46E-03	1.33E-03	7.78E-03
	15	3.93E-01	4.23E-03	3.97E-01	5.27E-02	1.08E-02	6.35E-02
	16	1.35E-02	1.45E-04	1.36E-02	1.81E-03	3.71E-04	2.18E-03
	17	1.96E-01	2.12E-03	1.98E-01	2.63E-02	5.41E-03	3.17E-02
	18	3.33E-02	3.60E-04	3.37E-02	4.47E-03	9.18E-04	5.39E-03
	19	1.02E-01	1.10E-03	1.03E-01	1.37E-02	2.81E-03	1.65E-02
	20	1.11E-02	1.20E-04	1.12E-02	1.49E-03	3.06E-04	1.80E-03
	21	7.78E-01	8.39E-03	7.86E-01	1.04E-01	2.14E-02	1.26E-01
	22	1.22E-03	1.32E-05	1.24E-03	1.64E-04	3.37E-05	1.98E-04
	23	1.44E-02	1.55E-04	1.45E-02	1.93E-03	3.96E-04	2.32E-03
	24	**1.08E+00**	1.17E-02	**1.09E+00**	1.45E-01	2.98E-02	1.75E-01
Thailand	1	7.41E-02	7.99E-04	7.49E-02	9.94E-03	2.04E-03	1.20E-02
	2	**1.05E+00**	1.13E-02	**1.06E+00**	1.41E-01	2.89E-02	1.69E-01
	3	**1.57E+00**	1.69E-02	**1.59E+00**	2.11E-01	4.33E-02	2.54E-01
	4	8.15E-02	8.79E-04	8.23E-02	1.09E-02	2.24E-03	1.32E-02
	5	5.92E-02	6.39E-04	5.99E-02	7.95E-03	1.63E-03	9.58E-03
	6	2.94E-01	3.17E-03	2.97E-01	3.94E-02	8.10E-03	4.75E-02
	7	3.59E-01	3.88E-03	3.63E-01	4.82E-02	9.89E-03	5.81E-02
	8	4.81E-02	5.19E-04	4.87E-02	6.46E-03	1.33E-03	7.78E-03
	9	1.33E-01	1.44E-03	1.35E-01	1.79E-02	3.67E-03	2.16E-02
	10	6.74E-03	7.27E-05	6.81E-03	9.04E-04	1.86E-04	1.09E-03
	11	3.54E-02	3.82E-04	3.58E-02	4.75E-03	9.76E-04	5.73E-03
	12	1.53E-02	1.65E-04	1.55E-02	2.05E-03	4.21E-04	2.47E-03
	13	1.43E-01	1.55E-03	1.45E-01	1.93E-02	3.95E-03	2.32E-02
	14	3.02E-02	3.26E-04	3.05E-02	4.05E-03	8.31E-04	4.88E-03
	15	9.62E-02	1.04E-03	9.73E-02	1.29E-02	2.65E-03	1.56E-02
	16	6.15E-01	6.63E-03	6.21E-01	8.25E-02	1.69E-02	9.94E-02
	17	**1.16E+00**	1.25E-02	**1.18E+00**	1.56E-01	3.20E-02	1.88E-01
	18	2.32E-01	2.50E-03	2.34E-01	3.11E-02	6.39E-03	3.75E-02
Vietnam	1	1.59E-01	1.72E-03	1.61E-01	2.14E-02	4.39E-03	2.57E-02
	2	**1.34E+00**	1.44E-02	**1.35E+00**	1.79E-01	3.68E-02	2.16E-01
	3	**1.26E+00**	1.36E-02	**1.27E+00**	1.69E-01	3.47E-02	2.04E-01

	4	5.92E-02	6.39E-04	5.99E-02	7.95E-03	1.63E-03	9.58E-03
	5	6.44E-02	6.95E-04	6.51E-02	8.64E-03	1.77E-03	1.04E-02
	6	1.25E-01	1.35E-03	1.27E-01	1.68E-02	3.45E-03	2.02E-02
	7	1.48E-02	1.60E-04	1.50E-02	1.99E-03	4.08E-04	2.40E-03
	8	1.52E-01	1.64E-03	1.53E-01	2.04E-02	4.18E-03	2.46E-02
	9	3.41E-01	3.68E-03	3.44E-01	4.57E-02	9.38E-03	5.51E-02
	10	6.67E-02	7.19E-04	6.74E-02	8.94E-03	1.84E-03	1.08E-02
	11	1.26E-01	1.36E-03	1.27E-01	1.69E-02	3.47E-03	2.04E-02
	12	1.22E-01	1.32E-03	1.24E-01	1.64E-02	3.37E-03	1.98E-02
	13	3.19E-01	3.44E-03	3.23E-01	4.28E-02	8.79E-03	5.16E-02
	14	1.00E-01	1.08E-03	1.01E-01	1.34E-02	2.75E-03	1.62E-02
	15	6.96E-01	7.51E-03	7.04E-01	9.34E-02	1.92E-02	1.13E-01
	16	6.30E-02	6.79E-04	6.36E-02	8.45E-03	1.73E-03	1.02E-02
	17	9.26E-02	9.99E-04	9.36E-02	1.24E-02	2.55E-03	1.50E-02
	18	9.63E-02	1.04E-03	9.73E-02	1.29E-02	2.65E-03	1.56E-02
	19	2.14E-02	2.31E-04	2.16E-02	2.87E-03	5.90E-04	3.46E-03
	20	4.44E-01	4.79E-03	4.49E-01	5.96E-02	1.22E-02	7.19E-02
	21	3.77E-02	4.06E-04	3.81E-02	5.05E-03	1.04E-03	6.09E-03
	22	2.59E-01	2.79E-03	2.62E-01	3.47E-02	7.13E-03	4.19E-02
	23	2.42E-01	2.61E-03	2.44E-01	3.24E-02	6.66E-03	3.91E-02
	24	3.15E-01	3.40E-03	3.19E-01	4.23E-02	8.69E-03	5.10E-02
East PM	1	2.03E-01	2.19E-03	2.05E-01	2.73E-02	5.60E-03	3.29E-02
	2	3.89E-02	4.19E-04	3.93E-02	5.22E-03	1.07E-03	6.29E-03
	3	4.33E-02	4.67E-04	4.38E-02	5.81E-03	1.19E-03	7.01E-03
	4	7.71E-02	8.32E-04	7.79E-02	1.03E-02	2.12E-03	1.25E-02
	5	1.64E-01	1.77E-03	1.66E-01	2.20E-02	4.52E-03	2.65E-02
	6	1.07E-03	1.16E-05	1.09E-03	1.44E-04	2.96E-05	1.74E-04
	7	7.59E-02	8.19E-04	7.67E-02	1.02E-02	2.09E-03	1.23E-02
	8	1.08E-01	1.17E-03	1.10E-01	1.46E-02	2.99E-03	1.75E-02
	9	5.92E-03	6.39E-05	5.99E-03	7.95E-04	1.63E-04	9.58E-04
	10	1.56E-01	1.69E-03	1.58E-01	2.10E-02	4.30E-03	2.53E-02
	11	2.43E-01	2.62E-03	2.45E-01	3.25E-02	6.68E-03	3.92E-02
	12	7.33E-02	7.91E-04	7.41E-02	9.84E-03	2.02E-03	1.19E-02
	13	8.37E-02	9.03E-04	8.46E-02	1.12E-02	2.31E-03	1.35E-02
	14	2.30E-01	2.49E-03	2.33E-01	3.09E-02	6.35E-03	3.73E-02
East Malaysia	1	1.52E-01	1.64E-03	1.53E-01	2.04E-02	4.18E-03	2.46E-02
	2	1.93E-01	2.08E-03	1.95E-01	2.58E-02	5.30E-03	3.11E-02
	3	3.89E-02	4.19E-04	3.93E-02	5.22E-03	1.07E-03	6.29E-03
	4	7.28E-02	7.86E-04	7.36E-02	9.77E-03	2.01E-03	1.18E-02
	5	1.98E-01	2.14E-03	2.01E-01	2.66E-02	5.47E-03	3.21E-02
	6	8.69E-02	9.38E-04	8.79E-02	1.17E-02	2.40E-03	1.41E-02
	7	9.74E-03	1.05E-04	9.84E-03	1.31E-03	2.68E-04	1.57E-03
	8	1.65E-02	1.78E-04	1.67E-02	2.21E-03	4.54E-04	2.66E-03
	9	1.21E-01	1.30E-03	1.22E-01	1.62E-02	3.33E-03	1.95E-02
	10	2.80E-02	3.02E-04	2.83E-02	3.75E-03	7.70E-04	4.52E-03
	11	2.59E-04	2.80E-06	2.62E-04	3.48E-05	7.14E-06	4.19E-05
Straits of Johore	1	1.14E-01	1.23E-03	1.15E-01	1.53E-02	3.14E-03	1.84E-02

	2	9.78E-02	1.05E-03	9.88E-02	1.31E-02	2.69E-03	1.58E-02
	3	2.59E-01	2.79E-03	2.62E-01	3.47E-02	7.13E-03	4.19E-02
	4	1.94E-01	2.10E-03	1.97E-01	2.61E-02	5.36E-03	3.15E-02
	5	1.24E-01	1.34E-03	1.26E-01	1.67E-02	3.43E-03	2.01E-02
	6	1.25E-01	1.35E-03	1.26E-01	1.67E-02	3.44E-03	2.02E-02
West PM	1	2.22E-02	2.40E-04	2.25E-02	2.98E-03	6.12E-04	3.59E-03
	2	1.30E-01	1.40E-03	1.31E-01	1.74E-02	3.57E-03	2.10E-02
	3	1.78E-01	1.92E-03	1.80E-01	2.38E-02	4.90E-03	2.87E-02
	4	7.70E-02	8.31E-04	7.79E-02	1.03E-02	2.12E-03	1.25E-02
	5	1.22E-01	1.32E-03	1.24E-01	1.64E-02	3.37E-03	1.98E-02
	6	1.67E-01	1.80E-03	1.68E-01	2.24E-02	4.59E-03	2.69E-02
	7	4.07E-02	4.39E-04	4.12E-02	5.47E-03	1.12E-03	6.59E-03
	8	1.33E-01	1.44E-03	1.35E-01	1.79E-02	3.67E-03	2.16E-02
	9	3.70E-04	4.00E-06	3.74E-04	4.97E-05	1.02E-05	5.99E-05
	10	4.33E-01	4.67E-03	4.38E-01	5.81E-02	1.19E-02	7.01E-02
	11	5.92E-02	6.39E-04	5.99E-02	7.95E-03	1.63E-03	9.58E-03
	12	8.22E-02	8.86E-04	8.31E-02	1.10E-02	2.26E-03	1.33E-02
	13	1.68E-01	1.81E-03	1.70E-01	2.25E-02	4.62E-03	2.71E-02
	14	1.33E-02	1.43E-04	1.34E-02	1.78E-03	3.66E-04	2.15E-03
	15	9.39E-02	1.01E-03	9.49E-02	1.26E-02	2.59E-03	1.52E-02
	16	3.55E-03	3.84E-05	3.59E-03	4.77E-04	9.79E-05	5.75E-04
	17	2.58E-01	2.79E-03	2.61E-01	3.47E-02	7.12E-03	4.18E-02
	18	6.11E-02	6.59E-04	6.18E-02	8.20E-03	1.68E-03	9.88E-03
	19	2.51E-01	2.71E-03	2.54E-01	3.37E-02	6.92E-03	4.06E-02
	20	2.08E-01	2.24E-03	2.10E-01	2.79E-02	5.72E-03	3.36E-02
	21	**1.50E+00**	1.61E-02	**1.51E+00**	2.01E-01	4.12E-02	2.42E-01
	22	2.13E-01	2.29E-03	2.15E-01	2.85E-02	5.86E-03	3.44E-02
	23	**4.69E+00**	5.06E-02	**4.74E+00**	6.29E-01	1.29E-01	7.59E-01
	24	9.63E-02	1.04E-03	9.73E-02	1.29E-02	2.65E-03	1.56E-02
	25	8.43E-01	9.10E-03	8.52E-01	1.13E-01	2.32E-02	1.36E-01
	26	1.12E-01	1.21E-03	1.13E-01	1.50E-02	3.08E-03	1.81E-02
	27	2.36E-01	2.54E-03	2.38E-01	3.16E-02	6.49E-03	3.81E-02
	28	2.25E-01	2.43E-03	2.28E-01	3.02E-02	6.20E-03	3.64E-02
	29	1.13E-01	1.21E-03	1.14E-01	1.51E-02	3.10E-03	1.82E-02
	30	2.31E-01	2.49E-03	2.33E-01	3.10E-02	6.36E-03	3.73E-02
	31	1.13E-02	1.22E-04	1.15E-02	1.52E-03	3.12E-04	1.83E-03
	32	3.03E-02	3.26E-04	3.06E-02	4.06E-03	8.33E-04	4.89E-03
	33	8.26E-02	8.91E-04	8.35E-02	1.11E-02	2.27E-03	1.34E-02
	34	2.96E-01	3.20E-03	2.99E-01	3.97E-02	8.16E-03	4.79E-02
	35	5.89E-02	6.35E-04	5.95E-02	7.90E-03	1.62E-03	9.52E-03
	36	2.28E-01	2.46E-03	2.31E-01	3.06E-02	6.28E-03	3.69E-02
	37	3.78E-02	4.07E-04	3.82E-02	5.07E-03	1.04E-03	6.11E-03
	38	4.67E-01	5.03E-03	4.72E-01	6.26E-02	1.29E-02	7.55E-02
	39	1.38E-01	1.49E-03	1.39E-01	1.85E-02	3.80E-03	2.23E-02
	40	1.00E-01	1.08E-03	1.01E-01	1.34E-02	2.75E-03	1.62E-02
	41	2.25E-01	2.43E-03	2.28E-01	3.02E-02	6.20E-03	3.64E-02
	42	8.33E-01	8.99E-03	8.42E-01	1.12E-01	2.30E-02	1.35E-01
	43	1.31E-01	1.42E-03	1.33E-01	1.76E-02	3.62E-03	2.13E-02
	44	1.13E-01	1.22E-03	1.14E-01	1.51E-02	3.11E-03	1.82E-02

	45	2.20E-01	2.38E-03	2.23E-01	2.95E-02	6.06E-03	3.56E-02
	46	8.72E-02	9.40E-04	8.81E-02	1.17E-02	2.40E-03	1.41E-02
	47	2.84E-01	3.06E-03	2.87E-01	3.81E-02	7.82E-03	4.59E-02
	48	5.75E-02	6.20E-04	5.81E-02	7.71E-03	1.58E-03	9.29E-03
	49	2.07E-03	2.24E-05	2.10E-03	2.78E-04	5.71E-05	3.35E-04
	50	5.78E-02	6.23E-04	5.84E-02	7.75E-03	1.59E-03	9.34E-03
	51	2.92E-01	3.15E-03	2.95E-01	3.92E-02	8.04E-03	4.72E-02
	52	2.37E-01	2.56E-03	2.40E-01	3.18E-02	6.54E-03	3.84E-02
	53	9.39E-02	1.01E-03	9.49E-02	1.26E-02	2.59E-03	1.52E-02
	54	6.39E-01	6.90E-03	6.46E-01	8.58E-02	1.76E-02	1.03E-01
	55	2.96E-02	3.20E-04	2.99E-02	3.97E-03	8.16E-04	4.79E-03
	56	9.52E-02	1.03E-03	9.62E-02	1.28E-02	2.62E-03	1.54E-02
	57	2.87E-02	3.09E-04	2.90E-02	3.85E-03	7.90E-04	4.64E-03
	58	2.34E-01	2.52E-03	2.36E-01	3.14E-02	6.44E-03	3.78E-02
	59	1.78E-01	1.92E-03	1.80E-01	2.39E-02	4.91E-03	2.88E-02
	60	2.38E-01	2.57E-03	2.41E-01	3.19E-02	6.56E-03	3.85E-02
	61	1.07E-01	1.15E-03	1.08E-01	1.43E-02	2.94E-03	1.72E-02
	62	3.29E-02	3.55E-04	3.32E-02	4.41E-03	9.06E-04	5.32E-03
	63	1.08E-01	1.17E-03	1.10E-01	1.45E-02	2.99E-03	1.75E-02
	64	3.27E-02	3.53E-04	3.31E-02	4.39E-03	9.01E-04	5.29E-03
	65	1.06E-01	1.14E-03	1.07E-01	1.42E-02	2.92E-03	1.71E-02
	66	1.38E-01	1.49E-03	1.40E-01	1.86E-02	3.82E-03	2.24E-02
	67	5.60E-02	6.04E-04	5.66E-02	7.51E-03	1.54E-03	9.05E-03
	68	2.20E-01	2.38E-03	2.23E-01	2.95E-02	6.07E-03	3.56E-02
Philippines	1	3.85E-02	4.15E-04	3.89E-02	5.17E-03	1.06E-03	6.23E-03
	2	2.22E-02	2.40E-04	2.25E-02	2.98E-03	6.12E-04	3.59E-03
	3	3.52E-01	3.80E-03	3.56E-01	4.72E-02	9.69E-03	5.69E-02
	4	1.59E-01	1.72E-03	1.61E-01	2.14E-02	4.39E-03	2.57E-02
	5	2.07E-01	2.24E-03	2.10E-01	2.78E-02	5.71E-03	3.35E-02
	6	2.70E-02	2.92E-04	2.73E-02	3.63E-03	7.45E-04	4.37E-03
	7	7.04E-02	7.59E-04	7.11E-02	9.44E-03	1.94E-03	1.14E-02
	8	3.22E-02	3.47E-04	3.25E-02	4.32E-03	8.86E-04	5.20E-03
	9	9.85E-02	1.06E-03	9.96E-02	1.32E-02	2.71E-03	1.59E-02
	10	7.55E-02	8.15E-04	7.64E-02	1.01E-02	2.08E-03	1.22E-02
	11	2.40E-01	2.59E-03	2.43E-01	3.22E-02	6.62E-03	3.89E-02
	ISQV-low	2.78E-01	3.00E-03	2.81E-01	3.73E-02	7.65E-03	4.49E-02
	ISQV-high	8.07E-01	8.71E-03	8.16E-01	1.08E-01	2.22E-02	1.31E-01
	ERL	1.73E-01	1.87E-03	1.75E-01	2.32E-02	4.76E-03	2.80E-02
	ERM	8.07E-01	8.71E-03	8.16E-01	1.08E-01	2.22E-02	1.31E-01
	TEL	1.12E-01	1.21E-03	1.13E-01	1.50E-02	3.08E-03	1.81E-02
	PEL	4.15E-01	4.48E-03	4.20E-01	5.57E-02	1.14E-02	6.72E-02
	TEL	1.11E-01	1.20E-03	1.12E-01	1.49E-02	3.06E-03	1.80E-02
	PEL	4.15E-01	4.47E-03	4.19E-01	5.56E-02	1.14E-02	6.71E-02
	SQG (TEC)	1.33E-01	1.43E-03	1.34E-01	1.78E-02	3.66E-03	2.15E-02
	SGQ (PEC)	4.74E-01	5.11E-03	4.79E-01	6.36E-02	1.31E-02	7.67E-02

	ISQG	1.12E-01	1.21E-03	1.13E-01	1.50E-02	3.08E-03	1.81E-02
	PEL	4.15E-01	4.47E-03	4.19E-01	5.56E-02	1.14E-02	6.71E-02
	1980	2.59E-01	2.80E-03	2.62E-01	3.48E-02	7.14E-03	4.19E-02
	2003	6.30E-02	6.79E-04	6.36E-02	8.45E-03	1.73E-03	1.02E-02
	1964	4.63E-02	4.99E-04	4.68E-02	6.21E-03	1.28E-03	7.49E-03
	1994	6.30E-02	6.79E-04	6.36E-02	8.45E-03	1.73E-03	1.02E-02
	2008	6.30E-02	6.79E-04	6.36E-02	8.45E-03	1.73E-03	1.02E-02
	1961	7.41E-02	7.99E-04	7.49E-02	9.94E-03	2.04E-03	1.20E-02
	1995	6.30E-02	6.79E-04	6.36E-02	8.45E-03	1.73E-03	1.02E-02
	2004	5.55E-02	5.99E-04	5.61E-02	7.45E-03	1.53E-03	8.98E-03

Table S5: Values of hazard quotient ingestion (HQ_{ing}), hazard quotient dermal (HQ_{dermal}) and hazard index (HI) of Zn for children and adults from the present study.

	No.	Children			Adult		
		HQ_{ing}	HQ_{dermal}	HI	HQ_{ing}	HQ_{dermal}	HI
Indonesia	1	2.62E-05	2.10E-07	2.64E-05	3.52E-06	5.36E-07	4.05E-06
	2	4.06E-04	3.25E-06	4.10E-04	5.45E-05	8.30E-06	6.28E-05
	3	3.49E-03	2.79E-05	3.51E-03	4.68E-04	7.12E-05	5.39E-04
	4	1.75E-03	1.40E-05	1.76E-03	2.35E-04	3.57E-05	2.70E-04
	5	5.33E-03	4.26E-05	5.37E-03	7.15E-04	1.09E-04	8.24E-04
	6	2.35E-03	1.88E-05	2.37E-03	3.15E-04	4.79E-05	3.63E-04
	7	5.49E-02	4.39E-04	5.54E-02	7.37E-03	1.12E-03	8.49E-03
	8	2.97E-03	2.38E-05	3.00E-03	3.99E-04	6.07E-05	4.59E-04
	9	4.15E-03	3.32E-05	4.18E-03	5.57E-04	8.48E-05	6.42E-04
	10	4.23E-03	3.38E-05	4.26E-03	5.67E-04	8.64E-05	6.54E-04
	11	2.67E-04	2.13E-06	2.69E-04	3.58E-05	5.44E-06	4.12E-05
	12	1.38E-03	1.10E-05	1.39E-03	1.85E-04	2.81E-05	2.13E-04
	13	3.81E-03	3.05E-05	3.84E-03	5.11E-04	7.78E-05	5.88E-04
	14	3.71E-03	2.97E-05	3.74E-03	4.98E-04	7.59E-05	5.74E-04
	15	3.69E-02	2.95E-04	3.72E-02	4.95E-03	7.54E-04	5.71E-03
	16	2.35E-03	1.88E-05	2.37E-03	3.16E-04	4.81E-05	3.64E-04
	17	2.17E-02	1.74E-04	2.19E-02	2.92E-03	4.44E-04	3.36E-03
	18	1.44E-03	1.15E-05	1.45E-03	1.93E-04	2.95E-05	2.23E-04
	19	8.15E-03	6.52E-05	8.22E-03	1.09E-03	1.67E-04	1.26E-03
	20	2.81E-03	2.24E-05	2.83E-03	3.76E-04	5.73E-05	4.34E-04
	21	4.94E-02	3.95E-04	4.98E-02	6.62E-03	1.01E-03	7.63E-03
	22	1.18E-02	9.47E-05	1.19E-02	1.59E-03	2.42E-04	1.83E-03
	23	2.98E-02	2.38E-04	3.00E-02	3.99E-03	6.08E-04	4.60E-03
	24	5.02E-04	4.02E-06	5.07E-04	6.74E-05	1.03E-05	7.77E-05
Thailand	1	1.53E-03	1.22E-05	1.54E-03	2.05E-04	3.12E-05	2.36E-04
	2	4.15E-03	3.32E-05	4.18E-03	5.57E-04	8.48E-05	6.42E-04
	3	1.09E-02	8.74E-05	1.10E-02	1.47E-03	2.23E-04	1.69E-03
	4	2.62E-03	2.10E-05	2.64E-03	3.52E-04	5.36E-05	4.05E-04
	5	3.06E-03	2.45E-05	3.08E-03	4.10E-04	6.25E-05	4.73E-04
	6	3.28E-03	2.62E-05	3.30E-03	4.40E-04	6.69E-05	5.07E-04
	7	3.93E-03	3.15E-05	3.96E-03	5.28E-04	8.03E-05	6.08E-04
	8	7.87E-04	6.29E-06	7.93E-04	1.06E-04	1.61E-05	1.22E-04
	9	5.55E-03	4.44E-05	5.59E-03	7.45E-04	1.13E-04	8.58E-04
	10	7.91E-04	6.33E-06	7.97E-04	1.06E-04	1.62E-05	1.22E-04
	11	4.08E-04	3.26E-06	4.11E-04	5.47E-05	8.33E-06	6.30E-05
	12	7.12E-05	5.70E-07	7.18E-05	9.56E-06	1.45E-06	1.10E-05
	13	3.45E-03	2.76E-05	3.48E-03	4.63E-04	7.05E-05	5.33E-04
	14	3.28E-04	2.62E-06	3.30E-04	4.40E-05	6.69E-06	5.07E-05
	15	2.03E-03	1.62E-05	2.05E-03	2.72E-04	4.14E-05	3.14E-04
	16	1.33E-03	1.06E-05	1.34E-03	1.78E-04	2.71E-05	2.05E-04
	17	1.74E-03	1.39E-05	1.75E-03	2.33E-04	3.55E-05	2.69E-04
	18	1.05E-02	8.39E-05	1.06E-02	1.41E-03	2.14E-04	1.62E-03
Vietnam	1	4.06E-03	3.25E-05	4.10E-03	5.45E-04	8.30E-05	6.28E-04
	2	2.16E-01	1.73E-03	2.18E-01	2.90E-02	4.42E-03	3.34E-02
	3	1.66E-02	1.33E-04	1.68E-02	2.23E-03	3.40E-04	2.57E-03

	4	1.75E-03	1.40E-05	1.76E-03	2.35E-04	3.57E-05	2.70E-04
	5	1.35E-03	1.08E-05	1.37E-03	1.82E-04	2.77E-05	2.09E-04
	6	4.02E-03	3.22E-05	4.05E-03	5.39E-04	8.21E-05	6.21E-04
	7	5.24E-04	4.19E-06	5.29E-04	7.04E-05	1.07E-05	8.11E-05
	8	4.11E-03	3.29E-05	4.14E-03	5.51E-04	8.39E-05	6.35E-04
	9	7.78E-03	6.22E-05	7.84E-03	1.04E-03	1.59E-04	1.20E-03
	10	2.84E-03	2.27E-05	2.86E-03	3.81E-04	5.80E-05	4.39E-04
	11	3.45E-03	2.76E-05	3.48E-03	4.63E-04	7.05E-05	5.34E-04
	12	2.79E-03	2.23E-05	2.81E-03	3.74E-04	5.69E-05	4.31E-04
	13	5.16E-03	4.13E-05	5.21E-03	6.93E-04	1.06E-04	7.98E-04
	14	1.75E-03	1.40E-05	1.76E-03	2.35E-04	3.57E-05	2.70E-04
	15	1.25E-02	1.00E-04	1.26E-02	1.68E-03	2.56E-04	1.94E-03
	16	3.19E-03	2.55E-05	3.22E-03	4.28E-04	6.52E-05	4.93E-04
	17	5.55E-03	4.44E-05	5.59E-03	7.45E-04	1.13E-04	8.58E-04
	18	4.94E-03	3.95E-05	4.98E-03	6.62E-04	1.01E-04	7.63E-04
	19	1.73E-04	1.38E-06	1.74E-04	2.32E-05	3.53E-06	2.67E-05
	20	2.15E-02	1.72E-04	2.17E-02	2.88E-03	4.39E-04	3.32E-03
	21	2.77E-04	2.22E-06	2.79E-04	3.72E-05	5.66E-06	4.28E-05
	22	5.21E-03	4.17E-05	5.25E-03	6.99E-04	1.06E-04	8.05E-04
	23	3.62E-03	2.90E-05	3.65E-03	4.86E-04	7.40E-05	5.60E-04
	24	9.26E-03	7.41E-05	9.34E-03	1.24E-03	1.89E-04	1.43E-03
East PM	1	3.17E-03	2.53E-05	3.19E-03	4.25E-04	6.47E-05	4.90E-04
	2	9.09E-04	7.27E-06	9.16E-04	1.22E-04	1.86E-05	1.41E-04
	3	9.74E-04	7.80E-06	9.82E-04	1.31E-04	1.99E-05	1.51E-04
	4	8.16E-04	6.53E-06	8.22E-04	1.09E-04	1.67E-05	1.26E-04
	5	4.06E-04	3.25E-06	4.10E-04	5.45E-05	8.30E-06	6.28E-05
	6	3.28E-05	2.62E-07	3.30E-05	4.40E-06	6.69E-07	5.07E-06
	7	2.68E-03	2.14E-05	2.70E-03	3.59E-04	5.47E-05	4.14E-04
	8	6.97E-03	5.57E-05	7.02E-03	9.34E-04	1.42E-04	1.08E-03
	9	9.39E-05	7.52E-07	9.47E-05	1.26E-05	1.92E-06	1.45E-05
	10	2.08E-03	1.66E-05	2.10E-03	2.79E-04	4.25E-05	3.22E-04
	11	2.77E-03	2.22E-05	2.80E-03	3.72E-04	5.67E-05	4.29E-04
	12	1.80E-03	1.44E-05	1.81E-03	2.41E-04	3.67E-05	2.78E-04
	13	1.44E-03	1.15E-05	1.45E-03	1.93E-04	2.94E-05	2.23E-04
	14	1.79E-03	1.43E-05	1.81E-03	2.41E-04	3.66E-05	2.77E-04
East Malaysia	1	1.79E-03	1.43E-05	1.81E-03	2.40E-04	3.66E-05	2.77E-04
	2	2.49E-03	1.99E-05	2.51E-03	3.34E-04	5.09E-05	3.85E-04
	3	7.82E-04	6.26E-06	7.88E-04	1.05E-04	1.60E-05	1.21E-04
	4	1.24E-03	9.90E-06	1.25E-03	1.66E-04	2.53E-05	1.91E-04
	5	1.97E-02	1.58E-04	1.99E-02	2.64E-03	4.03E-04	3.05E-03
	6	2.68E-03	2.14E-05	2.70E-03	3.60E-04	5.48E-05	4.14E-04
	7	9.61E-04	7.69E-06	9.69E-04	1.29E-04	1.96E-05	1.49E-04
	8	6.99E-04	5.59E-06	7.05E-04	9.38E-05	1.43E-05	1.08E-04
	9	3.03E-03	2.42E-05	3.05E-03	4.06E-04	6.19E-05	4.68E-04
	10	2.40E-03	1.92E-05	2.42E-03	3.22E-04	4.91E-05	3.72E-04
	11	2.74E-04	2.19E-06	2.76E-04	3.68E-05	5.60E-06	4.24E-05
Straits of Johore	1	1.14E-03	9.12E-06	1.15E-03	1.53E-04	2.33E-05	1.76E-04

	2	2.99E-03	2.39E-05	3.02E-03	4.02E-04	6.11E-05	4.63E-04
	3	1.01E-02	8.07E-05	1.02E-02	1.35E-03	2.06E-04	1.56E-03
	4	9.20E-03	7.36E-05	9.27E-03	1.23E-03	1.88E-04	1.42E-03
	5	7.87E-03	6.29E-05	7.93E-03	1.06E-03	1.61E-04	1.22E-03
	6	5.99E-03	4.79E-05	6.03E-03	8.03E-04	1.22E-04	9.25E-04
West PM	1	3.50E-04	2.80E-06	3.52E-04	4.69E-05	7.14E-06	5.40E-05
	2	3.32E-03	2.66E-05	3.35E-03	4.46E-04	6.78E-05	5.13E-04
	3	4.37E-03	3.50E-05	4.40E-03	5.86E-04	8.93E-05	6.76E-04
	4	3.21E-03	2.57E-05	3.24E-03	4.31E-04	6.56E-05	4.96E-04
	5	4.80E-03	3.84E-05	4.84E-03	6.44E-04	9.80E-05	7.42E-04
	6	6.12E-02	4.89E-04	6.17E-02	8.21E-03	1.25E-03	9.46E-03
	7	1.70E-03	1.36E-05	1.72E-03	2.29E-04	3.48E-05	2.63E-04
	8	3.98E-03	3.18E-05	4.01E-03	5.33E-04	8.12E-05	6.15E-04
	9	8.74E-05	6.99E-07	8.81E-05	1.17E-05	1.79E-06	1.35E-05
	10	2.11E-02	1.69E-04	2.13E-02	2.83E-03	4.31E-04	3.26E-03
	11	2.40E-03	1.92E-05	2.42E-03	3.22E-04	4.91E-05	3.72E-04
	12	1.32E-03	1.06E-05	1.33E-03	1.78E-04	2.70E-05	2.05E-04
	13	2.24E-02	1.79E-04	2.26E-02	3.01E-03	4.58E-04	3.47E-03
	14	1.75E-04	1.40E-06	1.76E-04	2.35E-05	3.57E-06	2.70E-05
	15	3.45E-03	2.76E-05	3.48E-03	4.63E-04	7.06E-05	5.34E-04
	16	1.36E-04	1.09E-06	1.37E-04	1.83E-05	2.78E-06	2.11E-05
	17	1.34E-02	1.07E-04	1.35E-02	1.79E-03	2.73E-04	2.07E-03
	18	1.88E-03	1.51E-05	1.90E-03	2.53E-04	3.85E-05	2.91E-04
	19	1.42E-02	1.13E-04	1.43E-02	1.90E-03	2.89E-04	2.19E-03
	20	7.38E-03	5.91E-05	7.44E-03	9.91E-04	1.51E-04	1.14E-03
	21	1.29E-02	1.03E-04	1.30E-02	1.74E-03	2.64E-04	2.00E-03
	22	1.44E-02	1.15E-04	1.45E-02	1.93E-03	2.95E-04	2.23E-03
	23	2.11E-02	1.69E-04	2.13E-02	2.84E-03	4.32E-04	3.27E-03
	24	2.19E-03	1.75E-05	2.21E-03	2.94E-04	4.48E-05	3.39E-04
	25	1.47E-02	1.17E-04	1.48E-02	1.97E-03	3.00E-04	2.27E-03
	26	1.39E-02	1.11E-04	1.40E-02	1.86E-03	2.83E-04	2.14E-03
	27	1.51E-03	1.21E-05	1.53E-03	2.03E-04	3.09E-05	2.34E-04
	28	2.32E-02	1.86E-04	2.34E-02	3.11E-03	4.74E-04	3.59E-03
	29	2.03E-03	1.62E-05	2.04E-03	2.72E-04	4.14E-05	3.13E-04
	30	9.04E-03	7.24E-05	9.12E-03	1.21E-03	1.85E-04	1.40E-03
	31	2.05E-03	1.64E-05	2.07E-03	2.75E-04	4.19E-05	3.17E-04
	32	3.40E-03	2.72E-05	3.42E-03	4.56E-04	6.94E-05	5.25E-04
	33	1.00E-03	8.04E-06	1.01E-03	1.35E-04	2.05E-05	1.55E-04
	34	4.30E-03	3.44E-05	4.33E-03	5.76E-04	8.77E-05	6.64E-04
	35	1.47E-03	1.17E-05	1.48E-03	1.97E-04	3.00E-05	2.27E-04
	36	1.39E-02	1.11E-04	1.40E-02	1.86E-03	2.83E-04	2.14E-03
	37	3.88E-03	3.10E-05	3.91E-03	5.20E-04	7.92E-05	5.99E-04
	38	2.11E-02	1.69E-04	2.13E-02	2.84E-03	4.32E-04	3.27E-03
	39	4.11E-03	3.29E-05	4.14E-03	5.51E-04	8.39E-05	6.35E-04
	40	2.24E-03	1.79E-05	2.26E-03	3.01E-04	4.58E-05	3.47E-04
	41	7.03E-03	5.63E-05	7.09E-03	9.44E-04	1.44E-04	1.09E-03
	42	2.84E-02	2.28E-04	2.87E-02	3.82E-03	5.81E-04	4.40E-03
	43	3.66E-03	2.93E-05	3.69E-03	4.91E-04	7.47E-05	5.65E-04
	44	1.57E-03	1.25E-05	1.58E-03	2.10E-04	3.20E-05	2.42E-04

	45	2.23E-03	1.78E-05	2.25E-03	2.99E-04	4.56E-05	3.45E-04
	46	2.68E-03	2.14E-05	2.70E-03	3.59E-04	5.47E-05	4.14E-04
	47	5.29E-03	4.23E-05	5.33E-03	7.10E-04	1.08E-04	8.18E-04
	48	1.30E-03	1.04E-05	1.31E-03	1.74E-04	2.65E-05	2.01E-04
	49	1.62E-05	1.29E-07	1.63E-05	2.17E-06	3.30E-07	2.50E-06
	50	2.10E-03	1.68E-05	2.12E-03	2.82E-04	4.29E-05	3.25E-04
	51	4.47E-02	3.58E-04	4.51E-02	6.00E-03	9.14E-04	6.92E-03
	52	1.19E-02	9.51E-05	1.20E-02	1.59E-03	2.43E-04	1.84E-03
	53	1.28E-03	1.03E-05	1.29E-03	1.72E-04	2.62E-05	1.98E-04
	54	5.69E-03	4.55E-05	5.74E-03	7.64E-04	1.16E-04	8.80E-04
	55	3.20E-03	2.56E-05	3.22E-03	4.29E-04	6.53E-05	4.94E-04
	56	5.48E-03	4.38E-05	5.52E-03	7.35E-04	1.12E-04	8.47E-04
	57	3.87E-04	3.10E-06	3.90E-04	5.19E-05	7.91E-06	5.98E-05
	58	4.51E-03	3.61E-05	4.55E-03	6.05E-04	9.21E-05	6.97E-04
	59	2.43E-03	1.94E-05	2.45E-03	3.26E-04	4.96E-05	3.75E-04
	60	3.70E-03	2.96E-05	3.73E-03	4.97E-04	7.56E-05	5.72E-04
	61	3.35E-03	2.68E-05	3.37E-03	4.49E-04	6.84E-05	5.17E-04
	62	1.32E-03	1.05E-05	1.33E-03	1.77E-04	2.69E-05	2.04E-04
	63	3.47E-03	2.78E-05	3.50E-03	4.66E-04	7.09E-05	5.36E-04
	64	9.41E-03	7.53E-05	9.49E-03	1.26E-03	1.92E-04	1.46E-03
	65	1.13E-02	9.05E-05	1.14E-02	1.52E-03	2.31E-04	1.75E-03
	66	9.83E-04	7.87E-06	9.91E-04	1.32E-04	2.01E-05	1.52E-04
	67	3.15E-03	2.52E-05	3.18E-03	4.23E-04	6.44E-05	4.87E-04
	68	1.33E-02	1.06E-04	1.34E-02	1.78E-03	2.71E-04	2.05E-03
Philippines	1	4.98E-03	3.99E-05	5.02E-03	6.68E-04	1.02E-04	7.70E-04
	2	2.62E-03	2.10E-05	2.64E-03	3.52E-04	5.36E-05	4.05E-04
	3	1.44E-02	1.15E-04	1.45E-02	1.93E-03	2.94E-04	2.22E-03
	4	5.72E-03	4.58E-05	5.77E-03	7.68E-04	1.17E-04	8.85E-04
	5	1.21E-02	9.65E-05	1.22E-02	1.62E-03	2.46E-04	1.86E-03
	6	2.18E-03	1.75E-05	2.20E-03	2.93E-04	4.46E-05	3.38E-04
	7	4.19E-03	3.36E-05	4.23E-03	5.63E-04	8.57E-05	6.48E-04
	8	3.26E-03	2.61E-05	3.29E-03	4.37E-04	6.66E-05	5.04E-04
	9	5.33E-03	4.26E-05	5.37E-03	7.15E-04	1.09E-04	8.24E-04
	10	4.89E-03	3.91E-05	4.93E-03	6.56E-04	9.99E-05	7.56E-04
	11	1.10E-02	8.77E-05	1.11E-02	1.47E-03	2.24E-04	1.69E-03
	ISQV-low	8.74E-03	6.99E-05	8.81E-03	1.17E-03	1.79E-04	1.35E-03
	ISQV-high	1.79E-02	1.43E-04	1.81E-02	2.40E-03	3.66E-04	2.77E-03
	ERL	6.55E-03	5.24E-05	6.61E-03	8.79E-04	1.34E-04	1.01E-03
	ERM	1.79E-02	1.43E-04	1.81E-02	2.40E-03	3.66E-04	2.77E-03
	TEL	5.42E-03	4.33E-05	5.46E-03	7.27E-04	1.11E-04	8.38E-04
	PEL	1.18E-02	9.47E-05	1.19E-02	1.59E-03	2.42E-04	1.83E-03
	TEL	5.42E-03	4.33E-05	5.46E-03	7.27E-04	1.11E-04	8.38E-04
	PEL	1.18E-02	9.47E-05	1.19E-02	1.59E-03	2.42E-04	1.83E-03
	SQG (TEC)	5.29E-03	4.23E-05	5.33E-03	7.09E-04	1.08E-04	8.17E-04
	SGQ (PEC)	2.01E-02	1.60E-04	2.02E-02	2.69E-03	4.10E-04	3.10E-03

	ISQG	5.42E-03	4.33E-05	5.46E-03	7.27E-04	1.11E-04	8.38E-04
	PEL	1.18E-02	9.47E-05	1.19E-02	1.59E-03	2.42E-04	1.83E-03
	1980	7.65E-03	6.12E-05	7.71E-03	1.03E-03	1.56E-04	1.18E-03
	2003	2.93E-03	2.34E-05	2.95E-03	3.93E-04	5.98E-05	4.53E-04
	1964	3.06E-03	2.45E-05	3.08E-03	4.10E-04	6.25E-05	4.73E-04
	1994	3.10E-03	2.48E-05	3.13E-03	4.16E-04	6.34E-05	4.80E-04
	2008	2.84E-03	2.27E-05	2.86E-03	3.81E-04	5.80E-05	4.39E-04
	1961	4.15E-03	3.32E-05	4.18E-03	5.57E-04	8.48E-05	6.42E-04
	1995	2.27E-03	1.82E-05	2.29E-03	3.05E-04	4.64E-05	3.51E-04
	2004	2.84E-03	2.27E-05	2.86E-03	3.81E-04	5.80E-05	4.39E-04
